# miR-451a is underexpressed and targets AKT/mTOR pathway in papillary thyroid carcinoma

**DOI:** 10.18632/oncotarget.7262

**Published:** 2016-02-08

**Authors:** Emanuela Minna, Paola Romeo, Matteo Dugo, Loris De Cecco, Katia Todoerti, Silvana Pilotti, Federica Perrone, Ettore Seregni, Luca Agnelli, Antonino Neri, Angela Greco, Maria Grazia Borrello

**Affiliations:** ^1^ Department of Experimental Oncology and Molecular Medicine, Molecular Mechanisms Unit, Fondazione IRCCS Istituto Nazionale dei Tumori, Milan, Italy; ^2^ Department of Experimental Oncology and Molecular Medicine, Functional Genomics Core Facility, Fondazione IRCCS Istituto Nazionale dei Tumori, Milan, Italy; ^3^ Laboratory of Pre-Clinical and Translational Research, IRCCS-CROB, Referral Cancer Center of Basilicata, Rionero in Vulture (PZ), Italy; ^4^ Department of Pathology, Fondazione IRCCS Istituto Nazionale dei Tumori, Milan, Italy; ^5^ Department of Diagnostic Imaging and Radiotherapy, Fondazione IRCCS Istituto Nazionale dei Tumori, Milan, Italy; ^6^ Department of Oncology and Hemato-Oncology, University of Milan, Milan, Italy; ^7^ Hematology Unit, Fondazione IRCCS Ca' Granda, Ospedale Maggiore Policlinico, Milan, Italy

**Keywords:** papillary thyroid carcinoma, miR-451a, miRNA, RET/PTC, AKT pathway

## Abstract

Papillary Thyroid Carcinoma (PTC) is the most frequent thyroid cancer. Although several PTC-specific miRNA profiles have been reported, only few upregulated miRNAs are broadly recognized, while less consistent data are available about downregulated miRNAs. In this study we investigated miRNA deregulation in PTC by miRNA microarray, analysis of a public dataset from The Cancer Genome Atlas (TCGA), literature review and meta-analysis based on a univocal miRNA identifier derived from miRBase v21. A list of 18 miRNAs differentially expressed between PTC and normal thyroid was identified and validated in the TCGA dataset. Furthermore, we compared our signature with miRNA profiles derived from 15 studies selected from literature. Then, to select possibly functionally relevant miRNA, we integrated our miRNA signature with those from two *in vitro* cell models based on the PTC-driving oncogene *RET/PTC1*. Through this strategy, we identified commonly deregulated miRNAs, including miR-451a, which emerged also by our meta-analysis as the most frequently reported downregulated miRNA. We showed that lower expression of miR-451a correlates with aggressive clinical-pathological features of PTC as tall cell variant, advanced stage and extrathyroid extension. In addition, we demonstrated that ectopic expression of miR-451a impairs proliferation and migration of two PTC-derived cell lines, reduces the protein levels of its recognized targets MIF, c-MYC and AKT1 and attenuates AKT/mTOR pathway activation.

Overall, our study provide both an updated overview of miRNA deregulation in PTC and the first functional evidence that miR-451a exerts tumor suppressor functions in this neoplasia.

## INTRODUCTION

Thyroid cancer (TC) is the most frequent malignancy of the endocrine system. Papillary thyroid carcinoma (PTC) is the prevalent histotype (approximately 80% of all TCs) and its incidence has steadily increased over the past 40 years [1, 2]. PTCs comprise several histological subtypes characterized by different morphology and prognosis; among these the classical, follicular and tall cell variants are the most common.

Most PTCs are effectively treated by surgical removal followed by adjuvant radioactive iodine (RAI) therapy and the 5-years survival is over the 95% [3]. Nevertheless, a fraction of patients do not respond to RAI therapy and/or progress to metastatic disease; in these cases the prognosis is poor and the 10-years survival drops to 10% [4]. Although several treatments have been tested in these patients, limited benefits have been achieved and effective therapies are still lacking [5].

Starting from the identification of the first oncogene in PTC, *RET/PTC1* [6], in almost 30 years of investigations several tumor-driving genetic events have been identified and characterized. A remarkable contribution in this sense has recently been made by the work of The Cancer Genome Atlas (TCGA) Research Network [7] that, by a multiplatform analysis of almost 500 PTCs, the largest cohort studied to date, extended and advanced the knowledge of the biology and the genomic landscape of this tumor. Their discoveries not only confirmed the well known drivers as *BRAF* (60%) and *RAS* (13%) mutations and *RET* and *NTRK* gene fusions (8.8%), but also identified additional PTC-driving alterations as novel gene fusions and mutations in *EIF1AX* gene as well as in gene involved in DNA repair, chromatin remodeling and PI3K/AKT pathway. Although TCGA findings led to a significant reduction of the fraction of PTCs with unknown genetic drivers (from 25% to less than 4%), the mechanisms underlying the development and progression of PTC remain to be fully elucidated.

Recent evidence indicated that in addition to genetic alterations PTC, like the majority of tumors, is characterized by aberrant expression of microRNAs (miRNAs), a class of small noncoding RNAs that regulate gene expression at post-transcriptional level. Since miRNAs are able to regulate multiple targets, their role in biological processes results simultaneously powerful and complex. In the last years many studies have investigated miRNA deregulation in PTC [8–21] and their utility as diagnostic and prognostic markers has already been suggested [22]. Overall in PTC, miRNA upregulation is well supported and specific miRNAs have been broadly recognized (e.g. miR-146b and miR-221/-222 cluster), whereas miRNA downregulation has been reported only by a subset of studies and with low consistency [23, 24]. Even though several functional studies have addressed the role of specific miRNAs in thyroid carcinogenesis [22, 24], the involvement of others remains unexplored. Further studies are thus required to better understand the consequences of miRNA deregulation in PTC as well as the molecular processes and networks in which these miRNAs operate.

miR-451a is located on chromosome 17q11.2 and its biogenesis occurs via a non-canonical pathway that depend on Ago2 protein [25]. miR-451a aberrant expression and role in tumor pathogenesis and development have already been reported in lung, breast, gastric and colorectal cancer, as well as in glioma and leukemia (reviewed in [25]), and more recently confirmed in many other types of cancers [26–32]. Moreover, in several malignancies it was also reported a significant association between low miR-451a expression and aggressive clinical-pathological features as lymph node metastases (LNM) [29, 32], dedifferentiation [29, 31], advanced TNM stage [29–31], metastases [26, 30], recurrence [26] and reduced overall survival [27, 30]. Several miR-451a validated targets have been reported (http://miRTarBase.mbc.nctu.edu.tw/) [33] including, among the others, MIF, c-MYC and AKT1.

In the present study we investigated miRNAs deregulation in PTC. We performed miRNA microarray analysis in a small proprietary series of PTCs and validated the identified miRNA signature in an independent and larger dataset publicly available from TCGA [7]. Furthermore, we carried out a literature review and meta-analysis and compared our miRNA signature with those derived from 15 published studies. Then, we combined our miRNA signature, with those derived from two *in vitro* cell models based on the PTC-driving oncogene *RET/PTC1* previously established by us [34]. Based on this analysis, we identified four consistently deregulated miRNAs: miR-222-3p, miR-199a-3p, miR-214-3p and miR-451a. Notably, miR-451a emerged also by our meta-analysis as the most frequently reported downregulated miRNA in PTC. Because the involvement of miR-451a has not been investigated in PTC so far, we focused on miR-451a attempting to explore its role in PTC.

## RESULTS

### miRNA expression profiles in PTC clinical samples

**Figure 1:**
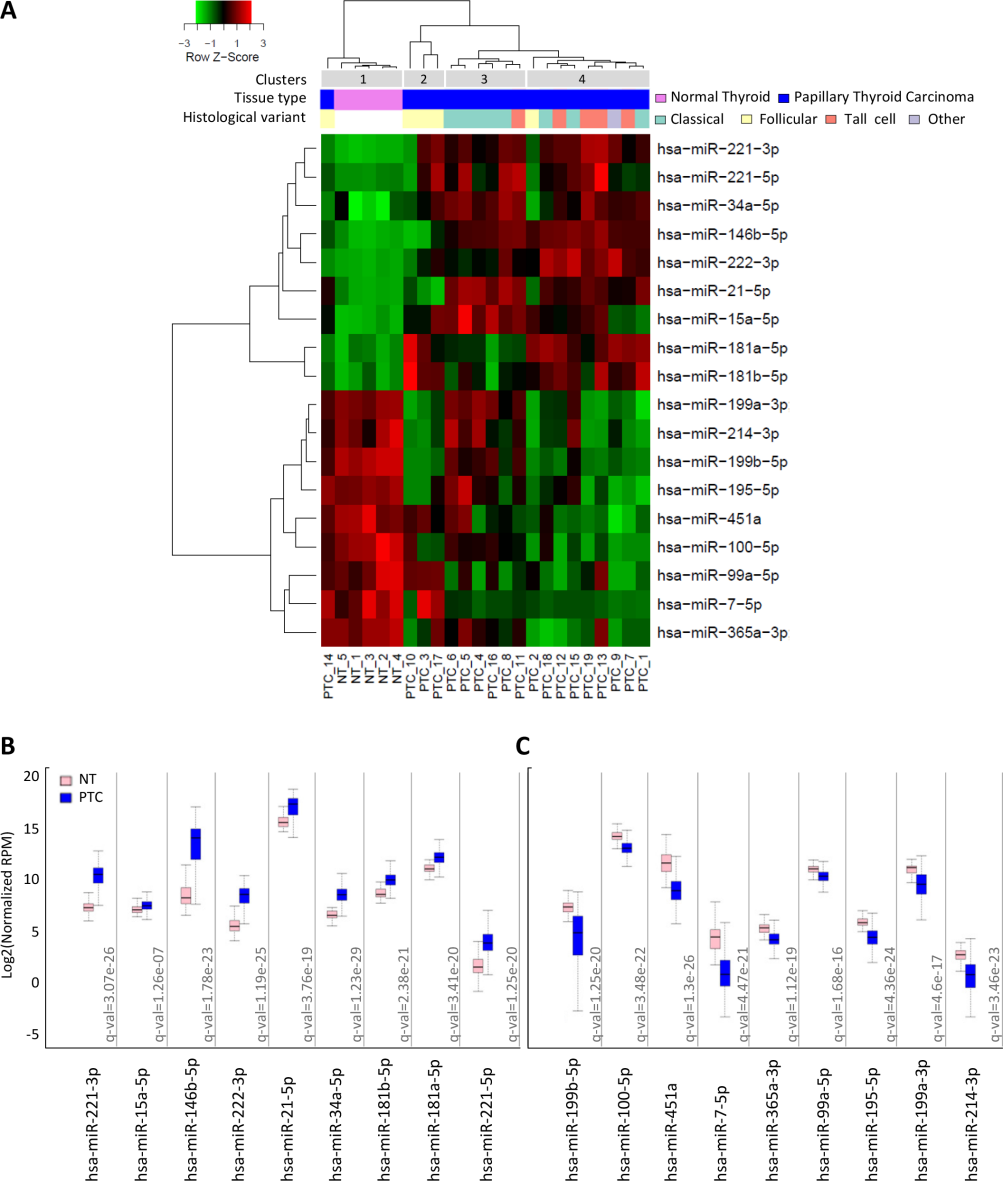
miRNA expression profiles in PTC clinical samples **A.** Heat map showing miRNAs differentially expressed between PTC and normal thyroid tissues (absolute FC≥1.5; FDR<0.05). **B-C.** miRNA expression in the validation set of 499 PTC and 59 normal thyroid tissues derived from TCGA. miR abundance is reported as log2 normalized (reads-per-million, RPM) median-centered; q-value, statistical significance calculated by Wilcoxon test adjusted by Benjamini-Hochberg correction.

miRNA expression was initially assessed by microarray in a series of 19 PTC and 5 normal thyroid tissues collected in our Institute (clinical-pathological features available in Supplemental Table S1). By class comparison analysis, we identified a list of 18 miRNAs significantly deregulated (absolute FC≥1.5; FDR<0.05) in PTC compared to normal thyroid (Supplemental Table S2); these included 9 upregulated miRNAs (miR-146b-5p, miR-221-3p, miR-222-3p, miR-21-5p, miR-34a-5p, miR-181a-5p, miR-15a-5p, miR-221-5p, miR-181b-5p) and 9 downregulated miRNAs (miR-451a, miR-7-5p, miR-199b-5p, miR-199a-3p, miR-195-5p, miR-100-5p, miR-365a-3p, miR-99a-5p, miR-214-3p). Hierarchical clustering analysis based on the identified miRNA list (Figure 1A), showed a clear separation between PTC and normal thyroid samples, as expected, and a partial sub-stratification of PTC samples according to histological type. Four major clusters were detected: cluster 1 including all normal thyroid samples (5/5; p = 0.0001); cluster 2 including follicular variant PTCs (3/5; p = 0.0049); cluster 3 enriched for classical variant PTCs (5/8; p = 0.0069) and cluster 4 enriched for tall cell variant PTCs (4/5; p = 0.0474).

As validation set, we took advantage of public data available from TCGA [7] and analyzed the expression of the 18 miRNAs in 499 PTCs and 59 normal thyroid samples. We confirmed the significant deregulation of both upregulated and downregulated miRNAs (Figure 1B–1C) in this independent and larger cohort. As further validation, hierarchical clustering analysis of the 59 matched PTC/adjacent normal thyroid samples included in TCGA dataset was performed based on the identified miRNAs list (Supplemental Figure S1). Our signature efficiently separated tumor samples from their normal counterpart also in this series of PTCs.

### Meta-analysis of miRNA expression profiles in PTC versus normal thyroid

**Figure 2:**
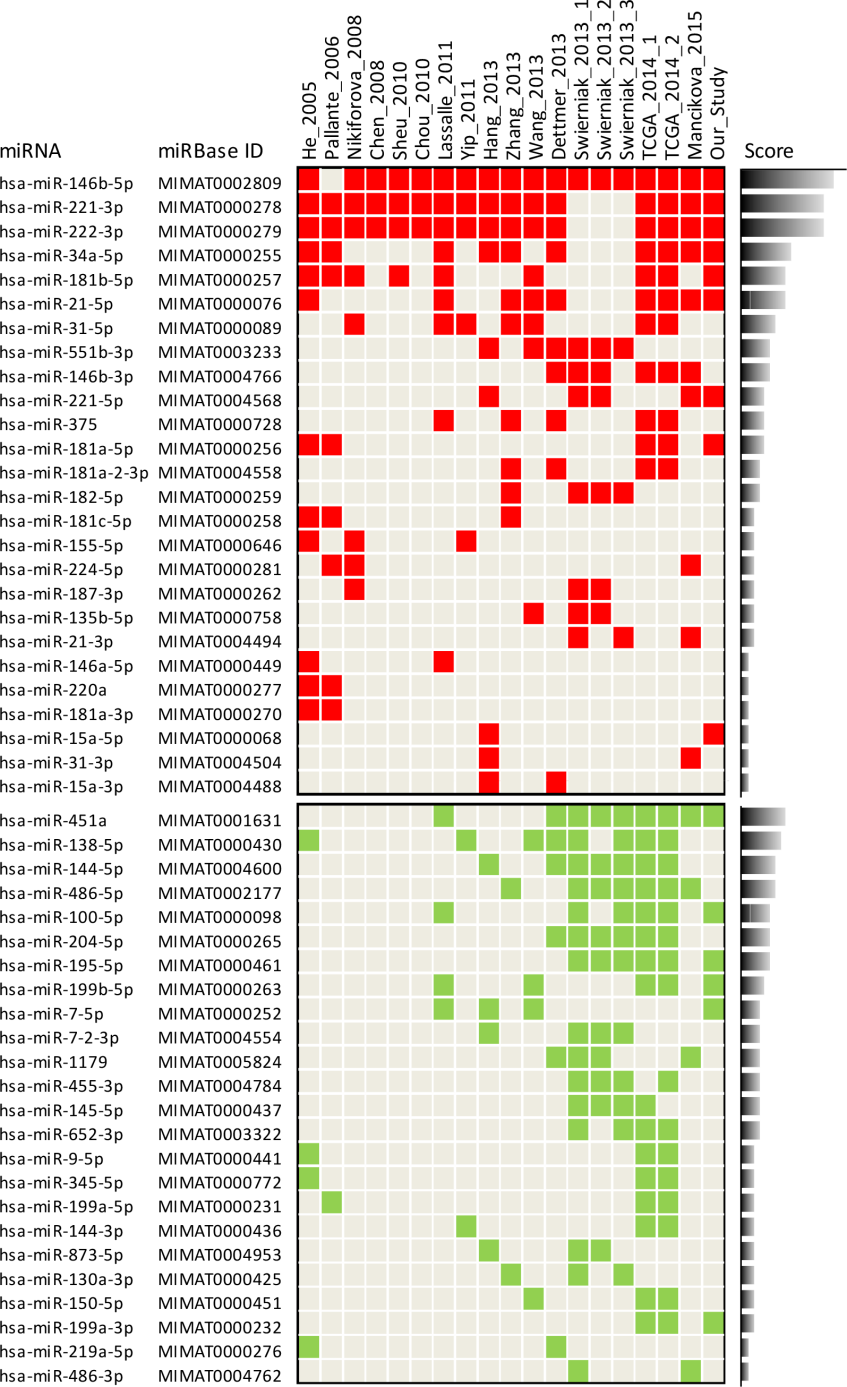
Meta-analysis of miRNA expression profiles in PTC versus normal thyroid Study-miRNA matrix showing miRNA expression across the 15 studies selected from literature and our study. Color code: red, upregulated; green, downregulated. Score is calculated based on the reporting frequency of each miRNA. Matrix selectively represents only miRNAs reported in at least two independent studies (complete data available in Supplemental Table S3); the three and two datasets derived from study 13 (Swierniak 2013) and from study 14 (TCGA 2014), respectively, were considered as single studies.

As previous studies have investigated miRNA deregulation in PTC, we explored to what extent our signature was consistent with literature data. We first carried out a systematic literature review and meta-analysis. To overcome the difficulties and heterogeneity associated with multiple datasets, we applied specific criteria of selection (see Materials and Methods) and focused only on studies reporting miRNA deregulation in PTC compared to normal thyroid tissue. Fifteen studies were selected from literature (Table 1). To assess the degree of concordance among studies, miRNA profiles were updated according to the most recent miRBase release (Supplemental Datasets and Supplemental Table S3; data processing fully described in Supplemental Materials and Methods). Overall we observed a common pattern of deregulation (Figure 2); in particular we confirmed better overlap across the studies for the upregulated than downregulated miRNAs, as already reported [23, 24]. Regarding upregulated miRNAs, along with miR-146b-5p, miR-221-3p and miR-222-3p, identified as top overexpressed in almost all studies, we found a set of miRNAs (miR-34a-5p, miR-181b-5p, miR-21-5p and miR-31-5p) consistently reported in at least half of the analyzed studies. Interestingly, a more in-depth analysis revealed that a number of the identified miRNAs belong to gene families (miR-221, miR-146 and miR-181) or clusters (miR-221/222 and miR-181a/181b) or derive from the same hairpin precursor (Supplemental Figure S2A).

**Table 1:** Overview of the 15 studies selected from literature

Study First author (year) [reference]	Specimens (type)	PTCs (number)	Normal thyroids (number)	miRNA profiling (method)
1 He (2005) [8]	snap-frozen	15	15, paired	microarray
2 Pallante (2006) [9]	snap-frozen	30	10, paired	microarray
3 Nikiforova (2008) [10]	snap-frozen	9	5	qRT-PCR panel
4 Chen (2008) [11]	FFPE	32	5	qRT-PCR
5 Sheu (2010) [12]	FFPE	10	10, paired	qRT-PCR
6 Chou (2010) [13]	snap-frozen	100	16, paired	qRT-PCR
7 Lassalle (2011) [14]	snap-frozen	16	16, paired	microarray
8 Yip (2011) [15]	snap-frozen	12	4	microarray
9 Huang (2013) [16]	snap-frozen	12	3, paired	microarray
10 Zhang (2013) [17]	snap-frozen	3	3, paired	microarray
11 Wang (2013) [18]	snap-frozen	6	2, paired	microarray
12 Dettmer (2013) [19]	FFPE	44	8	qRT-PCR panel
13 Swierniak_1 (2013) [20]	snap-frozen	14	14, paired	NGS
Swierniak_2 (2013) [20]	snap-frozen	14	14	NGS
Swierniak_3 (2013) [20]	snap-frozen	9	9, paired	microarray
14 TCGA_1 (2014) [7]	snap-frozen	59	59, paired	NGS
TCGA_2 (2014) [7]	snap-frozen	499	59, paired	NGS
15 Mancikova (2015) [21]	snap-frozen	78	17	NGS
Our study	snap-frozen	19	5	microarray

Regarding downregulated miRNAs, our meta-analysis revealed a set of miRNAs commonly reported, especially in the most recent studies; these included the most frequently identified miR-451a, miR-138-5p, miR-144-5p and miR-486-5p, and, reported with a lower frequency, miR-100-5p, miR-204-5p, miR-195-5p, miR-199b-5p, and miR-7-5p. In addition, also among downregulated miRNAs we could identify miRNAs included either in gene families (miR-7, miR-199 and miR-15) or clusters (miR-451a/144 and miR-7-2/1179) or derived from the same precursor (Supplemental Figure S2B).

When we compared our signature with those derived from literature, we observed a consistent overlap (Figure 2 and Supplemental Figure S2). In addition, our signature identified two further relevant miRNAs: miR-214-3p, included in the miR-199a-2/214 cluster, and miR-99a-5p, included along with miR-100-5p, in the miR-10 gene family (Supplemental Figure S3).

### Combined analysis of miRNAs in PTC clinical samples and *in vitro* cell models

**Figure 3:**
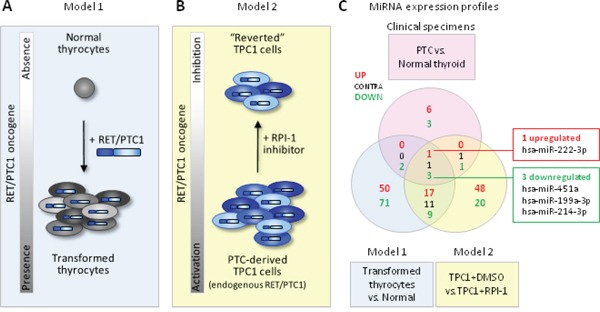
Combined analysis of miRNAs in PTC clinical samples and *in vitro* cell models **A-B.** Schematic representation of the two complementary *in vitro* cell models based on the PTC-driving oncogene *RET/PTC1*. Model 1: human primary thyrocytes exogenously expressing *RET/PTC1*; Model 2: PTC-derived cell line TPC1 expressing endogenous *RET/PTC1* treated with the RET inhibitor RPI-1. **C.** Venn diagram of miRNAs differentially expressed in clinical specimens (PTC vs. normal thyroid, absolute FC ≥1.5; FDR<0.05; see Supplemental Table S2) and in cell models (Model 1: *RET/PTC1*-expressing thyrocytes vs. parental thyrocytes, absolute FC ≥2.5; Model 2: TPC1 treated with solvent DMSO vs. TPC1 treated with RPI-1, absolute FC ≥2.5 and FDR <0.1). Common miRNAs concordantly expressed in the 3 datasets are indicated in the right boxes.

With the aim of selecting from our signature those miRNAs that could be functionally relevant and represent the best candidate for further analyses, we combined our miRNA signature with those derived from two *in vitro* cell models previously established by us [34] (Figure 3). The two models are based on the PTC-driving oncogene *RET/PTC1* and include primary human thyrocytes exogenously expressing RET/PTC1 (Model 1; Figure 3A) and the PTC-derived cell line TPC1, harboring endogenous *RET/PTC1*, treated with the RPI-1 inhibitor (Model 2; Figure 3B). The rationale for this analysis derived from the notion that in PTC different tumor-driving alterations converge on common signaling pathways (namely MAPK and PI3K/AKT pathway) [7], and that the use of these two cell models has already been helpful in the identification of functionally relevant miRNAs in PTC [34].

When we compared the three signatures, we found four consistently and concordantly deregulated miRNAs, including the upregulated miR-222-3p and the downregulated miR-451a, miR-199a-3p and miR-214-3p (Figure 3C). Of note, all these miRNAs appear relevant as: (i) the overexpression of miR-222-3p has broadly been reported (Figure 2) and is considered a hallmark of thyroid malignancy; (ii) the downregulation of miR-199a-3p and miR-214-3p, both included in the cluster miR-199a-2/214, has already been showed by us [34]; and (iii) miR-451a has been identified by our meta-analysis as the most frequently reported downregulated miRNA (Figure 2). We next focused on miR-451a as its involvement in various malignancies has already been reported, but in the context of PTC its functional role has not been investigated before.

### miR-451a expression relative to clinical pathological features and genetic lesions

**Figure 4:**
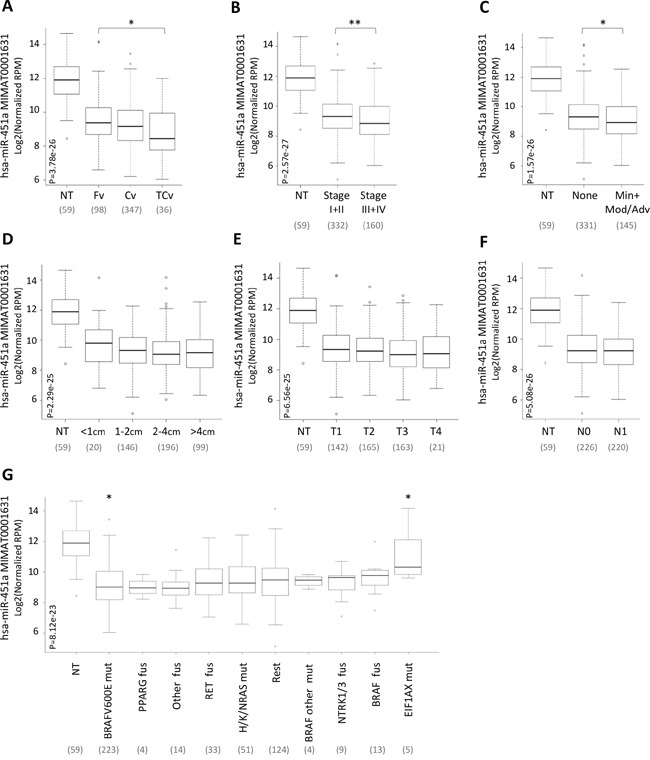
miR-451a expression relative to clinical pathological features and genetic lesions miR-451a expression in the set of 59 normal thyroid (NT) and 499 PTC samples derived from TCGA and stratified according to **A.** histological type (Fv, Follicular variant; Cv, Classic variant; TCv, Tall Cell variant), **B.** pathological TNM stage, **C.** extrathyroid extension, ETE (None, absence of ETE; Min, Minimal; Mod/Adv, Moderate/Advanced), **D.** tumor size, **E.** T stage, **F.** N stage (N0, absence of lymph node metastases; N1 presence of lymph node metastases) and **G.** genetic lesion (mut, mutation; fus, fusion; Rest, samples harboring other genetic lesions). miR-451a abundance is reported as log2 normalized (reads-per-million, RPM) and represented by box plots. In parentheses the number of samples included in each group. P, statistical significance by Kruskal-Wallis test. * p-value<0.05, ** p-value<0.01 by Wilcoxon test.

Starting from the notion that in various types of tumors low expression of miR-451a significantly correlates with aggressive clinical-pathological features, we tested whether a similar correlation existed also in PTC. As our PTC series was too small for appropriate samples stratification, we exploited once again the large cohort of PTC/normal thyroid samples of TCGA (Figure 4).

PTC samples were stratified based on the following clinical-pathological features: histological type (A), pathological TNM stage (B), extrathyroid extension (ETE) (C), tumor size (D), T stage (E) and N stage (lymph node metastases) (F); classification based on M stage was not applicable in the tested case list as only a few samples (8/499) presented distant metastases.

For all the tested parameters we observed that miR-451a expression was lower in each tumor subgroup than in normal thyroids (p-value <0.0001). Among PTCs, miR-451a expression was significantly lower in samples characterized by a more aggressive variant (tall cell; Figure 4A), advanced stage (stage III or IV; Figure 4B) and presence of ETE (Figure 4C). By contrast, no significant differences were found based on tumor size, T and N stage (Figure 4D–4F).

Then, the expression of miR-451a was further investigated in relation to different genetic lesions (Figure 4G). PTC samples were stratified based on various genetic lesions derived from the original study from TCGA [7] including: (i) mutations of *BRAF, RAS* and *EIF1AX* genes, (ii) fusions of *RET, NTRK, PPARG, BRAF* and “other genes” and (iii) group termed “Rest” comprising samples with other genetic lesions.

We found that the expression of miR-451a was significantly lower in each molecular subgroup of PTC than in normal thyroids (p-value <0.005), with the exception of *EIF1AX* mutated samples (p-value =0.27). Among PTCs we observed similar expression of miR-451a and significant differences were observed only in samples with *BRAF*V600E and *EIF1AX* mutations (p-value <0.05), showing lower and higher levels of miR-451a, respectively.

Overall these data, showing that miR-451a is significantly underexpressed in PTC and is differentially expressed according to specific clinical-pathological features and genetic lesions, prompted us to further investigate its functional role in the context of PTC.

### miR-451a functional studies

**Figure 5:**
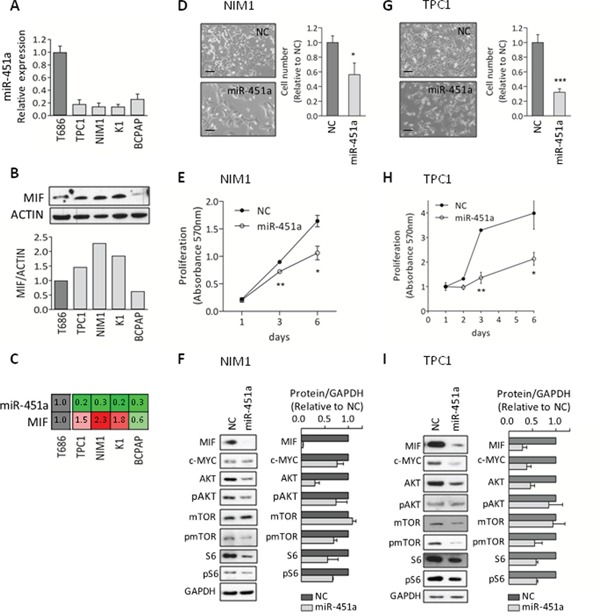
miR-451a functional studies **A.** miRNA-451a expression by qRT-PCR in the PTC-derived cell lines TPC1, NIM1, K1 and BCPAP, and in the normal thyroid control cells T686. Data are presented relative to the value of T686 cells. **B.** MIF protein expression by western blot analysis (WB) in the same panel of cell lines; Actin is shown as loading control. Below the corresponding densitometric quantification; MIF expression was normalized to Actin and presented relative to the level of T686 cells. **C.** Relative expression of the pair miR-451a and MIF; data are derived from the analyses reported in (A) and (B). **D-I.** Functional studies in NIM1 and TPC1 cells either transfected with miR-451a synthetic mimic (miR-451a) or Negative-Control (NC). **D, G.** Representative images of transfected cells (LEICA inverted microscope, scale bar 100μm). Cell number was determined by nuclei staining; data are presented relative to NC-transfected cells. **E, H.** Cell proliferation following transfection. **F, I.** WB analysis (day 3 after transfection). Protein expression was quantified and normalized to the loading control GAPDH. Data are presented relative to NC-transfected cells. Graphs report mean ± s.e.m. of at least two independent experiments. * p-value<0.05, ** p-value<0.005, *** p-value<0.0001 by Student's t-test.

Firstly, we evaluated the expression of miR-451a in a set of PTC-derived cell lines including TPC1, NIM1, K1 and BCPAP (Figure 5A). In agreement with data obtained in PTC specimens, we found that miR-451a was markedly downregulated in all the tested cell lines compared with the control cells T686. As several experimentally validated targets of miR-451a have been identified [33] and among these MIF is the most consistently reported (miRTarBase ID MIRT000046), we assessed MIF basal expression in the same panel of cell lines (Figure 5B). We observed that MIF protein was expressed at higher level in three out of the four tested cell lines compared with the control cell T686. Analyzing MIF expression in relation to miR-451a (Figure 5C), we found a general pattern of anticorrelation (3/4 of the tested cell lines), according to the expected inverse relationship between a miRNA and its target gene. Based on this result, we selected for the subsequent analyses NIM1 and TPC1 cell lines that, harboring *BRAF*V600E mutation and *RET/PTC1* rearrangement, respectively [35, 36], are representative of the mutation and gene fusion most frequently identified in PTC.

The functional role of miR-451a was investigated in both cell lines by the transfection of a miR-451a synthetic miRNA mimic. We verified in both cell lines that following transfection, miR-451a was efficiently overexpressed and concomitantly MIF protein level was strongly decreased (Supplementary Figure S4A). In functional assays we found that the ectopic expression of miR-451a significantly impaired cell growth, assessed by cell number count (Figure 5D and 5G) and proliferation assay (Figure 5E and 5H), and moderately reduced migratory ability (Supplementary Figure S4B-C).

To better understand the biological effects induced by miR-451a, we performed biochemical analyses (Figure 5F and 5I). In both cell lines we found that the ectopic expression of miR-451a was associated not only with the expected reduction of the validated targets MIF, c-MYC and AKT but also with decreased phosphorylation of AKT downstream effectors such as mTOR and S6 proteins. In detail, marked reduction was observed for MIF (mean reduction of 96% and 71% in NIM and TPC1 cells, respectively), AKT (mean reduction of 71% and 54%) and c-MYC (mean reduction of 24% and 62%) proteins. In addition, reduced levels of the phosphorylated proteins AKT (mean reduction of 26% and 15%), mTOR (mean reduction of 31% and 45%) and S6 (mean reduction of 33% and 41%) were detected. A clear reduction of total S6 protein (mean reduction of 44% and 42%) was also observed.

Collectively, these results suggest that in PTC-derived cell lines miR-451a displays tumor suppressor functions and targets multiple elements of the AKT/mTOR pathway.

## DISCUSSION

In this study we identified a panel of deregulated miRNAs in PTC specimens compared to normal thyroid and among these we showed that miR-451a is underexpressed and displays tumor suppressor functions by targeting multiple elements of the AKT/mTOR pathway. Our experimental strategy included: miRNA profiling, validation in an independent dataset from TCGA, literature review and meta-analysis, combined analysis of PTC clinical samples and *in vitro* cell models and functional studies.

Firstly, by miRNA profiling we identified a panel of 18 miRNAs significantly deregulated in PTC compared to normal thyroid. This miRNA signature was able both to separate tumor samples from normal thyroid and to partially sub-stratify PTC samples according to histological variants. The most significant separation was observed for the follicular variant that form an individual cluster separate from the other PTCs. This is consistent with previous reports [7, 19, 21] that highlighted how follicular variant PTCs represent a peculiar entity among PTCs sharing features with both PTC and follicular thyroid carcinoma (FTC). In addition, we identified two further clusters enriched for the classical (cluster 3) and for the tall cell variant (cluster 4), even though more heterogeneity, especially in the tall cell-enriched group (cluster 4), was found. However, due to intrinsic heterogeneity and to possible co-presence of different histological patterns in the same tumor, a complete separation among distinct histotypes may not occur as already reported [10], also in larger PTC cohorts [7, 19, 21].

As in the last decade several studies had already investigated miRNA expression in PTC, we compared our miRNA signature with those published. We performed a literature review; for a more homogeneous comparison, we applied stringent criteria of selection and we specifically focused only on studies using normal thyroid samples as healthy control group. However, a direct studies comparison resulted difficult, mostly because in some instances was applied heterogeneous miRNA nomenclature specific of different releases of miRBase. To overcome this problem, the identity of each miRNA was checked and updated according to miRBase (v21) mature sequence accession (i.e. MIMAT accession); this univocal ID was then used for a direct studies comparison. To our knowledge, this is the first report, among those analyzing miRNA profiles in PTC, that exploited this approach. Furthermore, for the first time, we included in our review the more recent publications in which miRNA expression was investigated by NGS [7, 20, 21].

Our meta-analysis not only underlined a set of upregulated miRNAs common to most studies [23, 24], but also identified a set of downregulated miRNAs consistently reported in the most recent studies. Recently, Riesco-Eizaguirre et al. determined by NGS miRNA deregulation in 8 matched PTC/normal thyroid samples [37]; their data are very consistent with those reported by our meta-analysis, providing further support to our results. Interestingly, some of the top downregulated miRNAs here identified, as miR-451a and miR-486-5p, had already been reported as unpublished data [38, 39]. Thus, our meta-analysis not only confirmed literature data but also shed new light on specific deregulated miRNAs whose possible relevance in PTC has been previously underestimated.

Along with the most frequently reported deregulated miRNAs, we identified several miRNAs reported only by a fraction of the analyzed studies. However, these miRNAs result of interest as they belong to specific miRNA families/clusters/precursors and thus appear in some way “linked” to each other. Indeed, miRNAs included in a gene family display sequence similarity, especially in the seed region, while miRNAs included in a cluster or precursor display physical proximity as they are transcribed in a single polycistronic transcript or derive from the same hairpin pre-miRNA, respectively. Collectively, these high related miRNAs are thought to be functionally related and to cooperate in the regulation of multiple biological processes either by co-targeting the same gene or different components of the same pathway. Interestingly, consistent patterns of deregulation have already been found in various tumors for these high related miRNAs [40]. Thus, the identification of concordant deregulation of miRNA families (e.g. miR-221,-146, -181, -199) or clusters (e.g. miR-221/222, -181a/181b, -451a/144, -7-2/1179) or mature miRNAs originated from the same precursor (e.g. miR-221-3p/5p, -146b-5p/3p, -21-5p/3p, -144-5p/3p, -199a-5p/3p, -486-5p/3p) in both up and downregulated miRNAs, suggests a major consistency for the their biological relevance in the pathology of PTC and provides a strong rationale to further investigate their role in this neoplasia by future studies.

The miRNA signature here identified, although derived from a small series of clinical samples, not only has been validated in an independent and larger cohort derived from TCGA, but also resulted consistent with literature data both in the general miRNA profile and in the coordinated deregulation of miRNA families/clusters/precursors.

Our meta-analysis identified miR-451a as the most frequently reported downregulated miRNA (Figure 2). In addition, miR-451a emerged also in the set of relevant miRNAs identified both in PTC clinical samples and *in vitro* cell models (Figure 3). These findings suggested the possible involvement of miR-451a in PTC and prompted us to focus on this miRNA.

In line with data reported in other tumors, we found a significant association between low expression of miR-451a and aggressive clinical-pathological features of PTC as tall cell variant, advanced TNM stage and presence of ETE, but not with tumor size, T and N stage. Recently, a systematic review [41] reported in PTC an association of miR-451a with lymph node metastases (LNM or also defined as N stage) and this may appear in contrast with our study. However, the association described by Aragon Han et al [41] was derived from a single study [42] and some considerations should be made about that study: first, miR-451a was reported moderately higher in patients with LNM (FC 1.6, P value 0.026) and this appear in contrast with data reported in other tumors [29, 32]; second the tested case list was smaller than that analyzed in the present study (87 and 446 PTC samples, respectively).

Among various genetic lesions we found significantly lower level of miR-451a in PTC patients with *BRAF*V600E mutation. Because in PTC this mutation has already been associated with poor clinical-pathological parameters [16, 19], this finding confirms the above mentioned association of miR-451a with aggressive features of PTC. Notably, a significant difference is also observed in PTCs with *EIF1AX* mutations in which miR-451a levels result higher if compared to the other PTC molecular subgroups.

*EIF1AX* encodes the translation initiation factor eIF1A that recently has been reported to promote the biogenesis of miR-451a by interacting with Ago2 protein [43]. The expression and the activity of eIF1A thus appear to be directly linked to mature miR-451a levels. In line with this notion, we found that EIF1AX is moderately but significantly underexpressed in PTC samples derived from TCGA (Supplemental Figure S5A); we could speculate that reduced expression of EIF1AX might cooperate or might be in part responsible of miR-451a downregulation in PTC. Our observation that PTCs with *EIF1AX* mutations express more miR-451a than PTCs with other genetic lesions (Figure 4G) is intriguing, although its significance remain to be determined. One hypothesis could be that these mutations might alter the eIF1A protein function and/or interaction with Ago2 and might promote a more effective processing and maturation of miR-451a. However, the functional effect of *EIF1AX* mutations is still unknown and further studies are required to test this hypothesis. As *EIF1AX* mutations have been recently found in various tumors [44–46], including thyroid tumors other than PTC [47], the identification of additional elements (e.g. miR-451a) linked to this gene is of general interest. However, as some *EIF1AX* mutations have been suggested to cause loss or impairment of protein function rather than gain [44, 46], we cannot exclude the possibility that additional mechanisms, not directly related to *EIF1AX* mutation, could contribute to the miR-451a higher expression in PTC patients with *EIF1AX* mutations.

The key role of miR-451a has already been demonstrated in various types of cancers. To our knowledge, its role in PTC has not been investigated before. Thus, to elucidate the processes and pathways regulated by miR-451a, we performed functional studies in PTC-derived cell lines. In agreement with previous reports [26–28, 31, 32], we found that the ectopic expression of miR-451a impairs cell proliferation and migration. Along with these biological effects, we observed the reduction at protein level of its validated targets MIF, c-MYC and AKT.

MIF is a pro-inflammatory cytokine overexpressed in various tumors, where it promotes tumor growth and progression by the activation of multiple signaling cascades, including AKT pathway [48]. Recently, its involvement has been described also in PTC. In detail, two independent studies showed that the pharmacological inhibition either of MIF [49] or its receptor CD74 [49, 50] impairs proliferation and migration of PTC-derived cell lines and reduces the activation of AKT/mTOR pathway. In line with these findings, here we showed that the inhibition of MIF protein expression by miR-451a, along with other target genes, impairs proliferation and migration. Interestingly, both studies [49, 50] described in PTC specimens a consistent overexpression of the MIF receptor and a trend toward MIF protein overexpression. According with these studies, we observed a moderate but significant overexpression of MIF in PTC samples derived from TCGA (Supplemental Figure S5B). Together these findings provide additional support for a role of MIF and its associated regulatory and signaling pathways, including miR-451a, in the context of PTC.

c-MYC is a well known transcription factor that integrating signals from multiple pathways, including AKT pathway, controls gene expression and cellular functions as cell proliferation, differentiation and transformation [51]. Importantly, *c-MYC* is an established oncogene and its aberrant activation occurs in many human cancers [51]. c-MYC deregulation has been found also in PTC where it is overexpressed at protein [52, 53] but not at mRNA level [52, 54, 55]. According to this, in PTC samples from TCGA we did not find c-MYC mRNA overexpression (Supplemental Figure S5C); by contrast, we found its moderate but significant underexpression. This finding, although unexpected, is in agreement with previous reports [52, 55] showing that c-MYC transcript level was lower in some thyroid tumors than in their normal counterpart. However, the significance of this finding remains to be elucidated.

As it is broadly recognized that miRNAs can regulate target genes by translation blockage without changes of mRNA level, we could hypothesize that in PTC miR-451a may regulate c-MYC by a similar mechanism. Interestingly, Kim et al. have recently proposed that in PTC c-MYC overexpression could be due to c-MYC protein stabilization induced by activated AKT signaling [52]. These findings thus suggest that in PTC c-MYC overexpression may occur by multiple mechanisms, including regulation by miRNAs (e.g. miR-451a) and post-translational events, that do not necessary involve mRNA level changes.

Here we showed that miR-451a mimic reduces both the c-MYC protein levels and the activation of AKT pathway (Figure 5F and 5I). As activated AKT pathway directly controls c-MYC protein levels [52], we cannot exclude the possibility that the observed reduction of c-MYC protein may be due to the combined action of miR-451a not only on c-MYC but also on its upstream regulators, namely on AKT. Indeed, AKT (specifically AKT1) is a target of miR-451a (miRTarBase ID MIRT005740) and consistently with this notion, we showed its reduction following miR-451a transfection.

AKT is a central mediator in the PI3K/AKT/mTOR pathway that in turn regulates fundamental cellular processes as proliferation and migration. Importantly, AKT is activated in many cancers, including thyroid carcinomas, where it is involved in tumor formation and progression [56]. Evidence of its activation has been reported also in PTC [7] and a trend toward AKT1 overexpression, both at mRNA and protein level, has already been described [57]. In line with these observations, we found a moderate but significant overexpression of AKT1 mRNA in PTC samples from TCGA (Supplemental Figure S5D).

In biochemical analyses, consistently with AKT reduction, we observed decreased phosphorylation of its downstream effectors mTOR and ribosomal protein S6, indicative of reduced pathway activation. Interestingly, we found also decreased expression of total S6 protein. However, this reduction may be due to feedback regulation rather than to a direct targeting by miR-451a. Indeed, S6 is not a reported target of miR-451a (according to miRTarBase v16) and we have previously showed its decrease following mTOR silencing [34]. Thus, we hypothesize that miR-451a by targeting AKT indirectly impairs the downstream activation of mTOR and this in turn causes S6 protein reduction.

Collectively, our functional analyses showed that in PTC, miR-451a affects cell proliferation and migration and targets multiple elements of the AKT/mTOR pathway, thus appearing to play a role as tumor suppressor miRNA in this neoplasia. We are aware that here we focused primarily on selected targets of miR-451a, already validated in other experimental sets, and that the identified link miR-451a/AKT pathway may represent only one, among many, of the molecular mechanisms by which this miRNA exerts its functions. Additional and more in-depth studies are thus required to fully elucidate the biological role of miR-451a in PTC. However, to our knowledge this is the first study investigating the functional role of miR-451a in PTC and the identification of a link miR-451a/AKT pathway in this tumor is noteworthy. Indeed, AKT pathway, along with MAPKs pathway, is a central hub in the signaling networks involved in thyroid carcinogenesis and several deregulated miRNAs have already been reported to target this pathway at multiple levels (reviewed in [23, 39]). Thus, the identification of miR-451a as an additional regulator of AKT pathway provides further evidence of the complexity of the molecular mechanisms involved in the control of this pathway crucial for both normal and neoplastic thyroid.

In summary, in this is work we gave a comprehensive and updated overview of the current knowledge about miRNA deregulation in PTC showing that along with the well known upregulated miRNAs a set of miRNAs emerges as consistently downregulated. In addition, we provided the first functional evidence that miR-451a displays tumor suppressor function in this neoplasia.

## MATERIALS AND METHODS

### Thyroid tissue samples

Nineteen PTC and five normal thyroid samples were obtained from the Department of Pathology, Fondazione IRCCS Istituto Nazionale dei Tumori (INT), Milan, Italy. PTC samples were classified according to WHO Classification [58] and to pathological tumor-node-metastasis (pTNM) staging system [59] by an expert pathologist; normal thyroid samples were obtained from patients with pathologies other than thyroid cancer. Informed consent was obtained by all patients whose biological samples were used in the study and the experimental protocol was approved by the Independent Ethical Committee of INT. Tumor samples were screened for the most common mutations and rearrangements reported in PTC including BRAF (exon 15), NRAS (exon 2) and HRAS (exon 2) mutations and RET and NTRK1 rearrangements. Detailed methods are described in Supplemental Materials and Methods.

### miRNA microarray analysis

Total RNA was extracted from snap-frozen tissues using the miRNeasy Mini kit (Qiagen). RNA concentration was measured with the NanoDrop ND-100 Spectrophotometer (NanoDrop Technologies, Wilmington, DE) and RNA quality was assessed with the Agilent 2100 Bioanalyzer (Agilent Technologies, Palo Alto, CA). RNA was labeled and processed according to the manufacturer's recommended protocol and miRNA expression analysis was assessed using SurePrint G3 Human miRNA 8x60K microarrays from Agilent Technologies. Briefly, 100ng of RNA were dephosphorylated with calf intestinal alkaline phosphatase and denatured in the presence of DMSO. Samples were fluorescently labeled with cyanine 3-pCp using T4 RNA ligase and hybridized on miRNA array. Arrays were washed in Agilent GE Wash Buffers and scanned at resolution of 2 mm using an Agilent DNA microarray scanner.

Data were acquired using Agilent's Feature Extraction software v10.7 and were analyzed using R programming language [60] and related Bioconductor [61] packages. Raw miRNA expression data were preprocessed using an optimized version of the RMA algorithm implemented in the *AgiMicroRna* package [62] and miRNAs detected in at least 15 samples (according to the *gIsGeneDetected* information given by the Feature Extraction software) were selected for further analyses. miRNAs differentially expressed between PTC and normal thyroid were identified using the *limma* package [63]. Multiple-testing correction was performed using the Benjamini-Hochberg false discovery rate (FDR) [64] and miRNAs with FDR < 0.05 and absolute fold-change ≥ 1.5 were considered significant. Clusters enrichment was determined by one-sided Fisher's exact test comparing each cluster with the others. Microarray data were deposited and are available on NCBI Gene Expression Omnibus (GEO) database (www.ncbi.nlm.nih.gov/geo/) with the accession number GSE73182.

### TCGA data analysis

miRNA datasets were retrieved from Illumina HiSeq Level 3 isoform quantification files [7] archived at the TCGA Data Portal website (http://tcga.cancer.gov/dataportal; accessed May 2015). The normalized reads per million miRNA mapped (RPM) data were obtained in 499 PTC and 59 normal thyroid samples by summing up the read counts at mature and star strand resolution for each MIMAT accession (miRBase v16), as described [65]. Mature miRNA species were then reannotated according to miRBase v21 (http://www.mirbase.org/) [66].

RPM data of the 18 miRNAs identified in this study were extracted both for 499 PTCs and 59 normal thyroids and for 59 matched PTC/normal thyroid cases. Data were log2 transformed after replacing the values equal to zero with the minimum non-null value. Differential miRNA expression between two groups was evaluated using Wilcoxon test. Adjustment for multiple testing was performed by Benjamini-Hochberg correction. Hierarchical clustering of the 59 matched PTC/normal thyroid samples was performed in DNAChip software [67] using pearson correlation and average as distance metric and linkage method, respectively, based on the expression profiles of our miRNA signature.

Differential expression of miR-451a in normal thyroid and PTC samples, stratified in multiple groups according to clinical-pathological features and genetic lesions [7], was evaluated using Kruskal Wallis test.

### Literature review and meta-analysis

A primary literature search was performed in PubMed (www.ncbi.nlm.nih.gov/pubmed) using the terms ‘thyroid cancer’ and ‘miRNA’ and all conceivable synonyms with limitations in ‘human’ and ‘English’ for paper published prior to July 31, 2015. Publications were considered eligible if they met the following criteria: (1) studies examining miRNAs in PTC, (2) studies examining miRNAs in thyroid cancers including PTC, (3) normal thyroid paired/unpaired used as healthy control group (4) miRNAs derived from tissue samples. Studies were excluded based on the following criteria: (1) reviews, single case reports, meta-analyses and abstract presented in conferences (2) absence of healthy control groups or use of healthy control groups other than normal thyroid as multinodular goiter, follicular adenomas or benign lesions (3) intra-tumoral comparisons as benign vs. malignant thyroid lesions or PTC not aggressive vs. aggressive (4) less than 3 miRNAs tested (5) circulating miRNAs assessment. According to our inclusion criteria, 15 studies were selected and full-text publications were reviewed in their entirety.

For meta-analysis and studies comparison, miRNA data were retrieved from the original publications and updated according to the most recent release of miRBase. In detail, the identity of each miRNA was verified by miRBase (v21) [66] and/or miRBase Tracker (www.mirbasetracker.org/index.php) [68] and an updated ID corresponding to MIMAT accession (miRBase v21) was assigned. A study-miRNA matrix was constructed using miRNA signature derived from each study and MIMAT accessions. Specific criteria of data filtering and cutoffs were applied for each study and are described in Supplemental Materials and Methods.

### *In vitro* models and cell cultures

The two *in vitro* cell models exploited in this study are based on *RET/PTC1* oncogene and were previously described [34]. The miRNA expression profiles derived from both models were deposited on GEO database (superSeries GSE49415) and have already been reported [34].

The cell lines TPC1, NIM1, K1 and BCPAP are derived from human PTC and have already been characterized for driving genetic lesions [35, 36]. The control cells T686 are derived from immortalized primary human non-neoplastic thyrocytes. Cell lines were cultured either in DMEM (TPC1, NIM1, and BCPAP) or DMEM: Ham's F12: MCDB at the ratio of 2:1:1 (K1 and T686) (Gibco, Thermo Fisher Scientific). All culture media were supplemented with 10% fetal bovine serum (EuroClone), and cells were maintained at 37°C and 5% CO_2_. Cell lines were authenticated by short tandem repeat (STR) profiles using the StemElite ID System (Promega) by the Fragment Analysis Facility at INT. Cells were routinely tested for mycoplasma.

### miR-451a expression in cultured cell lines

The endogenous expression of miR-451a in TPC1, NIM1, K1, BCPAP and T686 cell lines was determined by two-step quantitative real-time PCR. Total RNA, including miRNA fraction, was extracted from cultured cells with miRNeasy mini kit (Qiagen) and reverse-transcribed with TaqMan MicroRNA Reverse Transcription Kit (Applied Biosystems, Thermo Fisher Scientific). cDNA was amplified using TaqMan MicroRNA Assays together with TaqMan Universal PCR Master Mix (Applied Biosystems) on ABI PRISM 7900HT Real-Time PCR system. Data were analyzed with SDS 2.4 and RQ Manager 1.2.1 software (Applied Biosystems) using the 2^−ΔΔCt^ method. U6-snRNA was used as endogenous control for RNA input normalization.

### Transfection and functional studies

The ectopic expression of miR-451a was obtained in NIM1 and TPC1 cells by the transfection of miR-451a synthetic miRNA mimic (PM10286 Applied Biosystems) at 100 nM by siIMPORTER Transfection Reagent (Millipore, Billerica, MA); FAM-labeled Negative Control#1 (AM17121 Applied Biosystems) was transfected as negative control. Following transfection both cell lines were evaluated for cell number and proliferation. Cell number was assessed by NucleoCounter system (ChemoMetec A/S, Denmark) as previously described [34]. Cell proliferation was assessed by crystal violet assay. Briefly, at the indicated time points transfected cells were first fixed with 10% formalin for 20 min and then stained with 0.1% crystal violet (Sigma-Aldrich, MO, USA) for 30 min. After stain removal and PBS washes, the dye was solubilized with 1% SDS and the absorbance was measured at 570nm by a microplate reader (TecanUltra, Tecan Trading AG, Switzerland).

### Western blot analysis and antibodies

Total protein extraction, SDS PAGE and Western blot analyses were performed as previously described [69]. The primary antibody c-MYC (#5605), pAkt (Ser473, #4060), mTOR (#2983), pmTOR (Ser2448, #5536), S6 (#2217) and pS6 (Ser235/236, #4858) are from Cell Signaling Technology (Cell Signaling Technology Inc., MA, USA); MIF (ab175189) from Abcam (Abcam Inc., Cambridge, UK); AKT specific for AKT1 (#610861) from BD Transduction Laboratories (BD Biosciences, NJ, USA); Actin (A2066) from Sigma-Aldrich and GAPDH (#sc-32233) from Santa Cruz Biotechnology (Santa Cruz Biotechnology Inc., CA, USA). Relative protein levels were quantified by Quantity One 4.6.6 software (Bio-Rad, Hercules, CA).

### Statistical analysis

Detailed statistical analyses are reported in the specific sections miRNA microarray and TCGA data analysis. For functional studies, statistical analyses and graphs were generated using GraphPad Prism version 5.02; comparisons between two groups were performed by two-tailed Student's t-test with unequal variance. p-value < 0.05 was considered statistically significant.

## SUPPLEMENTARY DATA FIGURES AND TABLES






