# Correction: miR-451a is underexpressed and targets AKT/mTOR pathway in papillary thyroid carcinoma

**DOI:** 10.18632/oncotarget.24557

**Published:** 2018-02-23

**Authors:** Emanuela Minna, Paola Romeo, Matteo Dugo, Loris De Cecco, Katia Todoerti, Silvana Pilotti, Federica Perrone, Ettore Seregni, Luca Agnelli, Antonino Neri, Angela Greco, Maria Grazia Borrello

**Affiliations:** ^1^ Department of Experimental Oncology and Molecular Medicine, Molecular Mechanisms Unit, Fondazione IRCCS Istituto Nazionale dei Tumori, Milan, Italy; ^2^ Department of Experimental Oncology and Molecular Medicine, Functional Genomics Core Facility, Fondazione IRCCS Istituto Nazionale dei Tumori, Milan, Italy; ^3^ Laboratory of Pre-Clinical and Translational Research, IRCCS-CROB, Referral Cancer Center of Basilicata, Rionero in Vulture (PZ), Italy; ^4^ Department of Pathology, Fondazione IRCCS Istituto Nazionale dei Tumori, Milan, Italy; ^5^ Department of Diagnostic Imaging and Radiotherapy, Fondazione IRCCS Istituto Nazionale dei Tumori, Milan, Italy; ^6^ Department of Oncology and Hemato-Oncology, University of Milan, Milan, Italy; ^7^ Hematology Unit, Fondazione IRCCS Ca’ Granda, Ospedale Maggiore Policlinico, Milan, Italy

**This article has been corrected:** The updated Grant information is provided below:

**GRANT SUPPORT**

This work was supported by Associazione Italiana per la Ricerca sul Cancro (AIRC, Grant IG10366 to M.G. Borrello and Grant IG13307 to E. Seregni), by Ministero della Salute (Ricerca Finalizzata 2013 *RF-2013-02354985*) and in part by funds obtained through an Italian law that allows taxpayers to allocate 0.5 percent share of their income tax contribution to a research institution of their choice.

Original article: Oncotarget. 2016; 7:12731-12747. https://doi.org/10.18632/oncotarget.7262

