# Supplementary material for: miR-451a is underexpressed and targets AKT/mTOR pathway in papillary thyroid carcinoma
Source: Oncotarget. 2016 Feb 8;7(11):12731–47. doi: 10.18632/oncotarget.7262 (PMC4914318; doi:10.18632/oncotarget.7262)
Supplement: Supplementary file 1 [file oncotarget-07-12731-s001.pdf]

SUPPLEMENTARY DATA

PTC tissue samples molecular analysis

Tumor samples were screened for the most common mutations and rearrangements reported in PTC. Hematoxylin and eosin stained slides of FFPE tissues were reviewed by an expert pathologist and used to guide the macro-dissection of adjacent unstained sections. Paraffin was removed by xylene extraction followed by ethanol wash. BRAF (exon 15), NRAS (exon 2) and HRAS (exon 2) mutations were tested on genomic DNA extracted by Qiamp FFPE DNA kit (Qiagen, Chatsworth, CA, USA) and amplified by PCR as previously described [1]. PCR products were subjected to direct sequencing using an ABI Prism 3500 DX Genetic Analyzer (Applied Biosystems, Foster City, CA, USA) and then analyzed by ChromasPro software.

RET and NTRK1 rearrangements were tested on total RNA extracted by MasterPure RNA Purification Kit (Epicentre Biotechnologies, Madison, WI), including a DNaseI treatment step. cDNA was synthetized with random hexamers and Superscript™ III Reverse Transcriptase (Invitrogen, Carlsbad, CA, USA). To detect rearrangements, samples were first analyzed for the expression of tyrosine kinase (TK) domain and then positive samples were further tested for the expression of extracellular (EC) domain; samples expressing TK domain but not EC domain were considered rearranged. The following primers were used: RET TK domain sense 5’-TGCAGCGAGGAGATGTACC-3’ and antisense 5’-CCAGGTCTTTGCTGATGTCC-3’; RET EC domain sense 5’-CACCGGCCTCCTCTACCTTA-3’ and antisense 5’-AGCCGCGGTTGCGG ACACTG-3’; NTRK1 TK domain sense 5’-CCTCCGATCCCATGGACCTGAT-3’ and antisense 5’-CCTGCGTGATGCAGTCGATT-3’; NTRK1 EC domain sense 5’-ACCCTCTGCACTGTTC TTGT-3’ and antisense 5’-ATTGGGCACCTGGACCTTC-3’.

Literature review and meta-analysis

miRNA datasets of the 15 selected studies were retrieved from the original publications and manually curated. The specific criteria of data selection and filtering applied to each study are herein detailed. MIMAT accessions were assigned according to the most recent release of miRBase (v21).

Study 1 by He et al. (Supplemental dataset 1): we selected the list of miRNAs reported in Table 1 of the original publication independently of fold change (FC) and Local false discovery rates (Local FDR). To assign MIMAT accessions, we obtained probes sequences of the used miRNA chip [2,3] and directly verified the mature miRNA sequences in miRBase. Because in the same study the authors demonstrated in validation analyses the overexpression of hsa-miR-146b-5p (MIMAT0002809) rather than hsa-miR-146a-5p (MIMAT0000449), initially identified by microarray, we decided to include also this miRNA in the analyzed dataset.

Study 2 by Pallante et al. (Supplemental dataset 2): we selected the list of miRNAs reported in Table 1 of the original publication independently of FC and statistical significance (*P*). Data were ordered by decreasing FC. To assign MIMAT accession, we obtained probes sequences of the used miRNA chip [2] and performed alignments by BLAST (blast.ncbi.nlm.nih.gov/Blast) to identify within the probes the specific active site corresponding to the mature miRNA sequences. Once identified the mature miRNA sequences, these were directly verified in miRBase.

In the subsequent studies as more information was available about miRNAs identity and mature miRNA species (-3p/-5p), MIMAT accessions were assigned directly based on the ID reported in miRBase and/or in miRBase Tracker.

Study 3 by Nikiforova et al. (Supplemental dataset 3): we selected the list of top ten upregulated miRNAs reported in Table 1 of the original publication, identified in papillary carcinoma (PC) compared with normal thyroid tissue.

Study 4 by Chen et al. (Supplemental dataset 4): we selected only the three miRNAs analyzed in the PTC samples compared with normal thyroid tissue.

Study 5 Sheu et al. (Supplemental dataset 5): we selected only miRNAs analyzed in PTC compared with normal thyroid tissue and resulted statistically significant (*P*-value <0.05), reported in Table 3 of the original publication.

Study 6 by Chou et al. (Supplemental dataset 6): we selected the three analyzed miRNAs.

Study 7 by Lassalle et al. (Supplemental dataset 7): we selected the list of miRNAs reported in Table 4 of the original publication. Because these miRNA were identifed in PTC samples classified according to variant and BRAF mutation (FV-PTC, wtC-PTC and mutC-PTC) but the authors showed that the three classes had very similar miRNA signatures displaying more quantitative than qualitative differences, we analyzed simultaneously the three classes. Data were ordered by decreasing $\log_2$(FC) and an absolute cutoff value of 0.7 was applied. Only miRNAs passing the cutoff value and common among the three classes were included in the final dataset.

Study 8 by Yip et al. (Supplemental dataset 8): we selected the list of miRNAs reported in Figure 1a of the original publication.

Study 9 by Huang et al. (Supplemental dataset 9): we selected the list of miRNAs reported in Figure 1 of the

original publication, already filtered by FC (absolute FC >2) and statistical significance (*P*<0.05).

Study 10 by Zhang et al. (Supplemental dataset 10): we selected the list of miRNAs reported in Table 1 of the original publication, already filtered by statistical significance (*P*<0.05).

Study 11 by Wang et al. (Supplemental dataset 11): we selected the list of miRNAs reported in Table 2 of the original publication.

Study 12 by Dettmer et al. (Supplemental dataset 12): we selected the list of miRNAs reported in Table 2 of the original publication. As for study 7, we analyzed simultaneously the two reported classes of PTC (FVPTC and Classic PTC). We filtered out miRNAs (n=4) differentially expressed between the two classes of PTC and selected only miRNA statistically significant (p-value <0.05) in both classes.

Study 13 by Swierniak et al.: as in this study multiple comparisons and approaches were used, we decided to include in our meta-analysis all the three reported analyses. 1) NGS analysis in PTC and paired normal tissues (Supplemental dataset 13_1): we selected the list of 44 miRNAs statistically significant (paired FDR T vs. N <0.05) reported in Table 1 of the original publication. 2) NGS analysis in PTC and unrelated noncancerous thyroid (Supplemental dataset 13_2): we selected among miRNAs reported in Table 1 of the original publication only miRNAs with statistical significance (FDR T vs. NN <0.05). 3) miRNA microarray in additional PTC and paired normal tissues (Supplemental dataset 13_3): we selected among miRNA reported in Supplemental Table 3 of the original publication only miRNA with concordant expression (fold T-N) between NGS and microarray data.

Study 14 by TCGA: as for study 13, we included in our meta-analysis the two NGS analyses reported in this study. 1) paired PTC/normal thyroid tissues (Supplemental dataset 14_1) and 2) PTC and normal thyroid tissues (Supplemental dataset 14_2). We selected the list of miRNAs reported in Supplemental Table S6 of the original publication. Data were filtered by FC (absolute FC >2) and statistical significance (dataset 14_1 BH adjusted p-value from the Wilcoxon paired test <0.05; dataset 14_2 BH adjusted p-value from the Wilcoxon unpaired test <0.05).

Study 15 by Mancikova (Supplemental dataset 15): although in this study were reported two distinct NGS analyses in two independent sets of PTC and normal thyroid, limited overlap between the two sets was found possibly due to different RNA extraction technique used, as proposed by the authors. Therefore, for our meta-analysis we selected only the list of miRNAs commonly deregulated in both the analyzed sets reported in Supplemental Table S4 of the original publication.

REFERENCES

1. Di BM, Pietrantonio F, Perrone F, Dotti KF, Lampis A, Bertan C, Beretta E, Rimassa L, Carbone C, Biondani P, Passalacqua R, Pilotti S, Bajetta E. Lack of KRAS, NRAS, BRAF and TP53 mutations improves outcome of elderly metastatic colorectal cancer patients treated with cetuximab, oxaliplatin and UFT. Target Oncol 2014;9:155–162.
2. Liu CG, Calin GA, Meloon B, Gamliel N, Sevignani C, Ferracin M, Dumitru CD, Shimizu M, Zupo S, Dono M, Alder H, Bullrich F, Negrini M, Croce CM. An oligonucleotide microchip for genome-wide microRNA profiling in human and mouse tissues. Proc Natl Acad Sci U S A 2004;101:9740–9744.
3. Croce, C.M. U.S. Patent 8,389,210 B2, 2013.

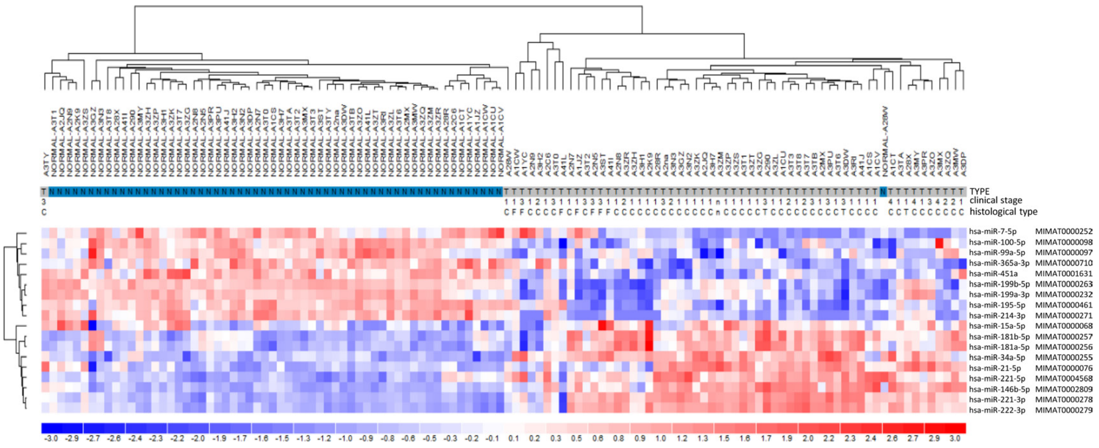

Supplemental Figure S1: Hierarchical clustering analysis based on our signature in the case list of 59 matched PTC/adjacent normal thyroid derived from TCGA.

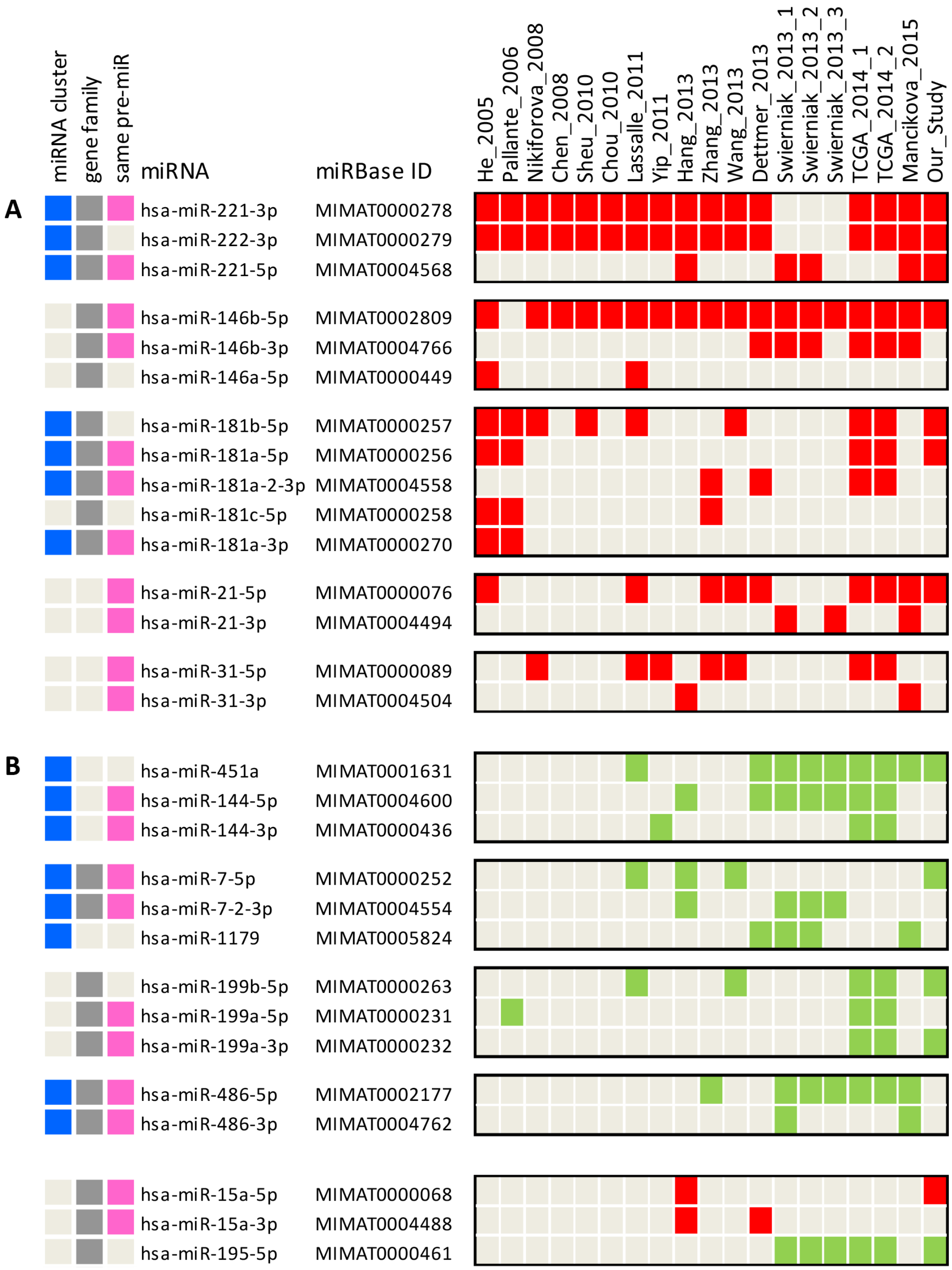

Supplemental Figure S2: Study-miRNA matrix showing the expression of specific miRNAs grouped according to clusters (blue), gene families (gray) and same pre-miR precursor (pink). Groups were assigned according to miRBase v21. Heatmap color code: red, upregulated (A); green, downregulated (B). Two members of miR-15 family display opposite expression pattern.

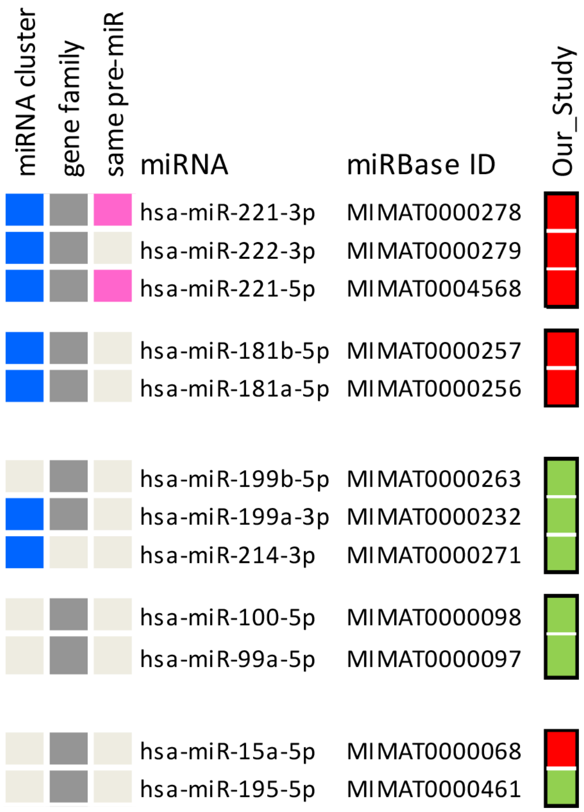

Supplemental Figure S3: Subset of miRNAs identified in the present study grouped according to clusters (blue), gene families (gray) and same pre-miR precursor (pink). Groups were assigned according to miRBase v21. Heatmap color code: red, upregulated (A); green, downregulated (B). Two members of miR-15 family display opposite expression pattern.

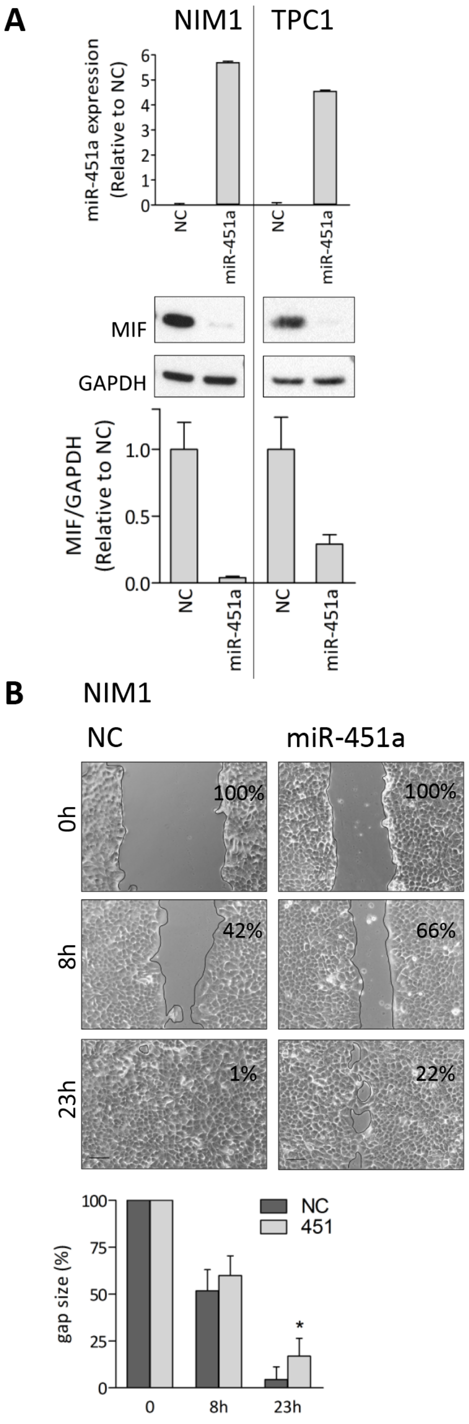

Supplemental Figure S4: A. miRNA-451a expression by qRT-PCR following the transfection of miR-451a synthetic mimic (miR-451a) or Negative-Control (NC); below the corresponding expression of MIF protein by WB and densitometric quantification normalized to the loading control GAPDH. RNA and protein samples were collected at 3 days post-transfection. **B-C.** Migration by wound healing assay. A wound was manually generated 2 days post-transfection and images were acquired at the indicated times by LEICA inverted microscope (scale bar NIM 100 µm; TPC1 250 µm). Gap size was calculated by Image-Pro Plus 7.0.1 software. Data are reported as mean ± s.e.m. for two independent experiments. *P value <0.05 determined by unpaired t-test.

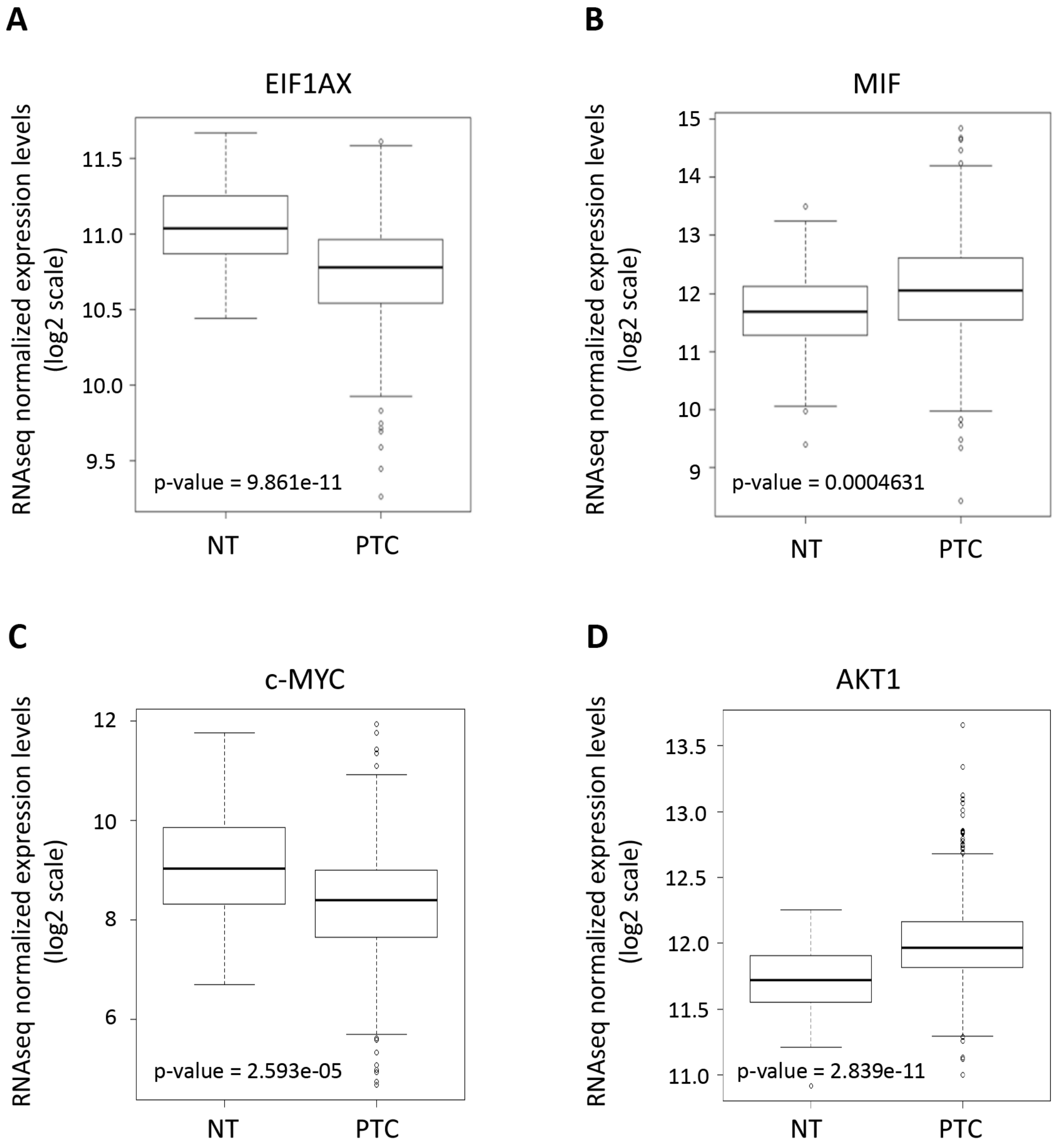

Supplemental Figure S5: Gene expression levels of EIF1AX **A.**, MIF **B.**, c-MYC **C.** and AKT1 **D.** in 58 normal thyroid (NT) and 486 PTC samples derived from TCGA. Normalized RNAseq expression values were derived from Illumina HiSeq Level 3 data, stored at the TCGA Data Portal website (http://tcga.cancer.gov/dataportal). Gene levels are represented by box plot distribution. p-value indicates differential expression between normal and tumor groups evaluated by Wilcoxon rank sum test.

Supplemental Table S1: PTC samples characterization

ID	sex	age	variant	T	N	M	stage	genetic lesion
PTC_1	F	37	Classical	2	1A	0	1	BRAF V600E
PTC_2	M	36	Follicular	3	X	0	1	Negative
PTC_3	F	51	Follicular	3	0	0	3	Negative
PTC_4	F	26	Classical	3	1A	0	1	RET rearr
PTC_5	F	38	Classical	3m	1B	0	1	Negative
PTC_6	M	46	Classical	3	1A	0	3	NA
PTC_7*	F	61	Tall cell	2m	1A	0	3	Negative
PTC_8	F	38	Classical	3	X	0	1	BRAF V600E
PTC_9*	M	52	Other	3m	X	0	3	HRAS Q61K
PTC_10	F	38	Follicular	2	X	0	1	NA
PTC_11*	F	48	Tall cell	3	1A	0	3	NA
PTC_12*	F	35	Tall cell	3m	1B	0	1	BRAF V600E
PTC_13*	F	51	Tall cell	3	0	0	2	BRAF V600E
PTC_14	F	52	Follicular	3	X	0	3	RET rearr
PTC_15/18**	M	35	Classical	3m	1A	0	1	Negative
PTC_16	F	33	Classical	3	1B	0	1	RET rearr
PTC_17	F	39	Follicular	3	X	0	1	NRAS Q61R
PTC_19	F	75	Tall cell	3m	1A	1	4C	Negative

Negative, negative for the tested genetic lesions including BRAF (exon15), N- HRAS(exon 2), RET and NTRK1 rearrengements; NA, not available; rearr, rearrangement.
* The original specimen from these patients contained areas showing PDTC component
** PTC_15 and PTC_18 are derived from the same patient and represent the primary tumor and lymph node metastasis, respectively; the reported clinicopathologic features are relative to the primary tumor.

Supplemental Table S2: Differentially expressed miRNAs in our series of PTCs

	miRNA_ID	miRBase21_ Accession	miRBase21_ID	Fold Change	FDR	Label
1	hsa-miR-146b-5p	MIMAT0002809	hsa-miR-146b-5p	18,17	0,0026	up
2	hsa-miR-221	MIMAT0000278	hsa-miR-221-3p	7,09	0,0008	up
3	hsa-miR-222	MIMAT0000279	hsa-miR-222-3p	4,65	0,0027	up
4	hsa-miR-21	MIMAT0000076	hsa-miR-21-5p	2,65	0,0078	up
5	hsa-miR-34a	MIMAT0000255	hsa-miR-34a-5p	2,36	0,0101	up
6	hsa-miR-181a	MIMAT0000256	hsa-miR-181a-5p	2,36	0,0221	up
7	hsa-miR-15a	MIMAT0000068	hsa-miR-15a-5p	2,08	0,0012	up
8	hsa-miR-221*	MIMAT0004568	hsa-miR-221-5p	1,93	0,0402	up
9	hsa-miR-181b	MIMAT0000257	hsa-miR-181b-5p	1,73	0,0200	up
10	hsa-miR-451	MIMAT0001631	hsa-miR-451a	-5,20	0,0101	down
11	hsa-miR-7	MIMAT0000252	hsa-miR-7-5p	-4,55	0,0200	down
12	hsa-miR-199b-5p	MIMAT0000263	hsa-miR-199b-5p	-3,78	0,0008	down
13	hsa-miR-199a-3p	MIMAT0000232	hsa-miR-199a-3p	-3,69	0,0479	down
14	hsa-miR-195	MIMAT0000461	hsa-miR-195-5p	-2,39	0,0479	down
15	hsa-miR-100	MIMAT0000098	hsa-miR-100-5p	-2,10	0,0011	down
16	hsa-miR-365	MIMAT0000710	hsa-miR-365a-3p	-1,83	0,0221	down
17	hsa-miR-99a	MIMAT0000097	hsa-miR-99a-5p	-1,76	0,0328	down
18	hsa-miR-214	MIMAT0000271	hsa-miR-214-3p	-1,62	0,0485	down

Supplemental Table S3:

See Supplementary File 1

Supplemental Datasets:

See Supplementary File 2
