# Supplementary material for: miR-451a is underexpressed and targets AKT/mTOR pathway in papillary thyroid carcinoma
Source: Oncotarget. 2016 Feb 8;7(11):12731–47. doi: 10.18632/oncotarget.7262 (PMC4914318; doi:10.18632/oncotarget.7262)
Supplement: Supplementary file 3 [file oncotarget-07-12731-s003.docx]

|  | **Supplemental Dataset 1.** | | | | | | | |  |
| --- | --- | --- | --- | --- | --- | --- | --- | --- | --- |
|  | miRNA_ID | mature miRNA  sequence (5′ to 3′) ^A^ | miRBase21  mature sequence | miRBase21  Accession | miRBase21_ID | FC | Local FDR (%) | Label |  |
| 1 | hsa-mir-146 | ugagaacugaauuccauggguu | UGAGAACUGAAUUCCAUGGGUU | MIMAT0000449 | hsa-miR-146a-5p | 19.3 | 4.4 | up |  |
| 2 | hsa-mir-221 | agcuacauugucugcuggguuuc | AGCUACAUUGUCUGCUGGGUUUC | MIMAT0000278 | hsa-miR-221-3p | 12.3 | 3.2 | up |  |
| 3 | hsa-mir-222* | agcuacaucuggcuacugggucuc | AGCUACAUCUGGCUACUGGGU | MIMAT0000279 | hsa-miR-222-3p | 10.9 | 2.7 | up |  |
| 4 | hsa-mir-21* | uagcuuaucagacugauguuga | UAGCUUAUCAGACUGAUGUUGA | MIMAT0000076 | hsa-miR-21-5p | 4.3 | 6.7 | up |  |
| 5 | hsa-mir-220 | ccacaccguaucugacacuuu | n.a (Dead miRNA entry MI0001386) | MIMAT0000277 | hsa-miR-220a | 4.0 | 8.6 | up |  |
| 6 | hsa-mir-181a | aacauucaacgcugucggugagu | AACAUUCAACGCUGUCGGUGAGU | MIMAT0000256 | hsa-miR-181a-5p | 2.6 | 6.9 | up |  |
| 7 | hsa-mir-181c | aacauucaaccugucggugagu | AACAUUCAACCUGUCGGUGAGU | MIMAT0000258 | hsa-miR-181c-5p | 2.4 | 7.6 | up |  |
| 8 | hsa-mir-181* | aacauucauugcugucgguggguu | AACAUUCAUUGCUGUCGGUGGGU | MIMAT0000257 | hsa-miR-181b-5p | 2.2 | 7.2 | up |  |
| 9 | hsa-mir-155 | uuaaugcuaaucgugauagggg | UUAAUGCUAAUCGUGAUAGGGGU | MIMAT0000646 | hsa-miR-155-5p | 2.2 | 9.1 | up |  |
| 10 | hsa-mir-213 | accaucgaccguugauuguacc | ACCAUCGACCGUUGAUUGUACC | MIMAT0000270 | hsa-miR-181a-3p | 1.9 | 7.3 | up |  |
| 11 | hsa-mir-34a | uggcagugucuuagcugguugu | UGGCAGUGUCUUAGCUGGUUGU | MIMAT0000255 | hsa-miR-34a-5p | 1.8 | 21.9 | up |  |
| 12 | hsa-mir-24-2 | uggcucaguucagcaggaacag | UGGCUCAGUUCAGCAGGAACAG | MIMAT0000080 | hsa-miR-24-3p | 1.7 | 10.1 | up |  |
| 13 | hsa-mir-29a-2 | cuagcaccaucugaaaucgguu | UAGCACCAUCUGAAAUCGGUUA | MIMAT0000086 | hsa-miR-29a-3p | 1.7 | 21.7 | up |  |
| 14 | hsa-mir-29b | uagcaccauuugaaaucagu | UAGCACCAUUUGAAAUCAGUGUU | MIMAT0000100 | hsa-miR-29b-3p | 1.7 | 23.7 | up |  |
| 15 | hsa-mir-29c | uagcaccauuugaaaucgguua | UAGCACCAUUUGAAAUCGGUUA | MIMAT0000681 | hsa-miR-29c-3p | 1.6 | 21.6 | up |  |
| 16 | hsa-mir-102 | uagcaccauuugaaaucagu | UAGCACCAUUUGAAAUCAGUGUU | MIMAT0000100 | hsa-miR-29b-3p | 1.6 | 11.2 | up |  |
| 17 | hsa-mir-24-1 | uggcucaguucagcaggaacag | UGGCUCAGUUCAGCAGGAACAG | MIMAT0000080 | hsa-miR-24-3p | 1.5 | 14.5 | up |  |
| 18 | hsa-mir-9-3 ^B^ | ucuuugguuaucuagcuguauga | UCUUUGGUUAUCUAGCUGUAUGA | MIMAT0000441 | hsa-miR-9-5p | 0.7 | 7.3 | down |  |
|  |  | uaaagcuagauaaccgaaagu | AUAAAGCUAGAUAACCGAAAGU | MIMAT0000442 | hsa-miR-9-3p |  |  | down |  |
| 19 | hsa-mir-219-1 | ugauuguccaaacgcaauucu | UGAUUGUCCAAACGCAAUUCU | MIMAT0000276 | hsa-miR-219a-5p | 0.6 | 5.7 | down |  |
| 20 | hsa-mir-138-1 | agcugguguugugaauc | AGCUGGUGUUGUGAAUCAGGCCG | MIMAT0000430 | hsa-miR-138-5p | 0.6 | 5.0 | down |  |
| 21 | hsa-mir-138-2 | agcugguguugugaauc | AGCUGGUGUUGUGAAUCAGGCCG | MIMAT0000430 | hsa-miR-138-5p | 0.6 | 4.9 | down |  |
| 22 | hsa-mir-345 | ugcugacuccuaguccagggc | GCUGACUCCUAGUCCAGGGCUC | MIMAT0000772 | hsa-miR-345-5p | 0.6 | 4.7 | down |  |
| 23 | hsa-mir-26a-1 | uucaaguaauccaggauaggcu | UUCAAGUAAUCCAGGAUAGGCU | MIMAT0000082 | hsa-miR-26a-5p | 0.6 | 5.0 | down |  |
| ^A^ retrieved from references [2,3] of Supplemental materials and methods; ^B^ two different mature miRNA sequences are reported in the precursor miR-9-3 [3] | | | | | | | | | |

|  |  | **Supplemental Dataset 2.** | | | | | | | | | |
| --- | --- | --- | --- | --- | --- | --- | --- | --- | --- | --- | --- |
|  |  | miRNA_ID | Oligo name ^A^ | Oligo sequence ^A^ | BLAST | miRBase21  Accession | miRBase21_ID | FC | *P* | Label |  |
| 1 | | miR-222 | hsa-miR-222-prec-#1 | CTTTCGTAATCAGCAGCTACATCTGGCTACTGGGTCTCTG | NR_029636.1 | MIMAT0000279 | hsa-miR-222-3p | 4,63 | 0.00256 | up |  |
| 2 | | miR-221 | hsa-miR-221-prec | CAACAGCTACATTGTCTGCTGGGTTTCAGGCTACCTGGAA | NR_029635.1 | MIMAT0000278 | hsa-miR-221-3p | 3,66 | 0.0143 | up |  |
| 3 | | miR-181b ^B^ | hsa-miR-181b-prec-#1 | TGAGGTTGCTTCAGTGAACATTCAACGCTGTCGGTGAGTT | NR_029626.1 | MIMAT0000256 | hsa-miR-181a-5p | 2,97 | 3.68E-05 | up |  |
|  | |  | hsa-miR-181b-prec-#2 | ACCATCGACCGTTGATTGTACCCTATGGCTAACCATCATC | NR_029626.1 | MIMAT0000270 | hsa-miR-181a-3p |  |  | up |  |
| 4 | | miR-220 | hsa-miR-220-prec | TGTGGCATTGTAGGGCTCCACACCGTATCTGACACTTTGG | AY866248.1 | MIMAT0000277 | hsa-miR-220a | 2,35 | 0.00435 | up |  |
| 5 | | miR-213 | hsa-miR-213-prec-#1 | AACATTCATTGCTGTCGGTGGGTTGAACTGTGTGGACAAG | NR_029612.1 | MIMAT0000257 | hsa-miR-181b-5p | 2,13 | 0.000595 | up |  |
| 6 | | miR-181a | hsa-miR-181a-prec-#1 | TCAGAGGACTCCAAGGAACATTCAACGCTGTCGGTGAGTT | NR_029611.1 | MIMAT0000256 | hsa-miR-181a-5p | 1,87 | 0.000247 | up |  |
| 7 | | miR-224 | hsa-miR-224-prec | GGCTTTCAAGTCACTAGTGGTTCCGTTTAGTAGATGATTG | NR_029638.1 | MIMAT0000281 | hsa-miR-224-5p | 1,79 | 0.0415 | up |  |
| 8 | | miR-181c | hsa-miR-181c-prec-#1 | TGCCAAGGGTTTGGGGGAACATTCAACCTGTCGGTGAGTT | NR_029613.1 | MIMAT0000258 | hsa-miR-181c-5p | 1,74 | 7.88E-05 | up |  |
| 9 | | miR-125a | hsa-miR-125a-prec-#1 | TCTAGGTCCCTGAGACCCTTTAACCTGTGAGGACATCCAG | NR_029693.1 | MIMAT0000443 | hsa-miR-125a-5p | 1,69 | 0.00207 | up |  |
| 10 | | miR-125b-1 | hsa-miR-125b-1 | TCCCTGAGACCCTAACTTGTGATGTTTACCGTTTAAATCC | NR_029671.1 | MIMAT0000423 | hsa-miR-125b-5p | 1,66 | 0.0471 | up |  |
| 11 | | miR-125b-2 | hsa-miR-125b-2-prec-#2 | ACCAGACTTTTCCTAGTCCCTGAGACCCTAACTTGTGAGG | NR_029694.1 | MIMAT0000423 | hsa-miR-125b-5p | 1,56 | 0.0364 | up |  |
| 12 | | miR-200a | hsa-miR-200a-prec | GTCTCTAATACTGCCTGGTAATGATGACGGCGGAGCCCTG | NR_029639.1 | MIMAT0000318 | hsa-miR-200b-3p | 1,42 | 0.00026 | up |  |
| 13 | | miR-196-2 | hsa-miR-196-2-prec-#2 | GCTGATCTGTGGCTTAGGTAGTTTCATGTTGTTGGGATTG | NR_029617.1 | MIMAT0000226 | hsa-miR-196a-5p | 1,38 | 0.0224 | up |  |
| 14 | | miR-034 | hsa-miR-034-prec-#1 | GAGTGTTTCTTTGGCAGTGTCTTAGCTGGTTGTTGTGAGC | NR_029610.1 | MIMAT0000255 | hsa-miR-34a-5p | 1,27 | 0.0143 | up |  |
| 15 | | miR-202 | hsa-miR-202-prec | GATCTGGCCTAAAGAGGTATAGGGCATGGGAAGATGGAGC | NR_030170.1 | MIMAT0002811 | hsa-miR-202-3p | 1,25 | 0.0275 | up |  |
| 16 | | miR-015-b | hsa-miR-015b-prec-#1 | GGCCTTAAAGTACTGTAGCAGCACATCATGGTTTACATGC | NR_029663.1 | MIMAT0000417 | hsa-miR-15b-5p | -1,2 | 0.00879 | down |  |
| 17 | | let-7f-1 | hsa-let-7f-1-prec-#2 | GGGATGAGGTAGTAGATTGTATAGTTGTGGGGTAGTGATT | NR_029483.1 | MIMAT0000067 | hsa-let-7f-5p | -1,21 | 0.0299 | down |  |
| 18 | | miR-148 | hsa-miR-148-prec | TGAGTATGATAGAAGTCAGTGCACTACAGAACTTTGTCTC | NR_029597.1 | MIMAT0000243 | hsa-miR-148a-3p | -1,27 | 0.0466 | down |  |
| 19 | | miR-199a-1 | hsa-miR-199a-1-prec | GCCAACCCAGTGTTCAGACTACCTGTTCAGGAGGCTCTCA | NR_029586.1 | MIMAT0000231 | hsa-miR-199a-5p | -1,32 | 0.00178 | down |  |
| 20 | | miR-140-as | hsa-miR-140-#2 | TTCTACCACAGGGTAGAACCACGGACAGGATACCGGGGCA | NR_029681.1 | MIMAT0004597 | hsa-miR-140-3p | -1,33 | 0.000948 | down |  |
| 21 | | miR-151 | hsa-miR-151-prec | GTATGTCTCATCCCCTACTAGACTGAAGCTCCTTGAGGAC | NR_029892.1 | MIMAT0000757 | hsa-miR-151a-3p | -1,42 | 0.00935 | down |  |
| 22 | | miR-142-as | hsa-miR-142-prec | CCCATAAAGTAGAAAGCACTACTAACAGCACTGGAGGGTG | NR_029683.1 | MIMAT0000433 | hsa-miR-142-5p | -1,46 | 0.00535 | down |  |
|  |  | ^A^ retrieved from reference [2] of Supplemental materials and methods; ^B^ two different oligos are reported in the precursor miR-181b [2]  In each oligo sequence is highlighted the active site corresponding to the mature miRNA sequence identified by BLAST alignment | | | | | | | | | |

|  | **Supplemental Dataset 3.** | | | | |
| --- | --- | --- | --- | --- | --- |
|  | miRNA_ID | miRBase21_Accession | miRBase21_ID | FC | Label |
| 1 | miR-187 | MIMAT0000262 | hsa-miR-187-3p | 73.7 | up |
| 2 | miR-221 | MIMAT0000278 | hsa-miR-221-3p | 19.1 | up |
| 3 | miR-222 | MIMAT0000279 | hsa-miR-222-3p | 17.2 | up |
| 4 | miR-181b | MIMAT0000257 | hsa-miR-181b-5p | 14.4 | up |
| 5 | miR-146b | MIMAT0002809 | hsa-miR-146b-5p | 10.5 | up |
| 6 | miR-155 | MIMAT0000646 | hsa-miR-155-5p | 9.5 | up |
| 7 | miR-122a | MIMAT0000421 | hsa-miR-122-5p | 8.9 | up |
| 8 | miR-31 | MIMAT0000089 | hsa-miR-31-5p | 7.5 | up |
| 9 | miR-205 | MIMAT0000266 | hsa-miR-205-5p | 6.8 | up |
| 10 | miR-224 | MIMAT0000281 | hsa-miR-224-5p | 6.2 | up |

|  | **Supplemental Dataset 4.** | | | |
| --- | --- | --- | --- | --- |
|  | miRNA_ID | miRBase21_Accession | miRBase21_ID | Label |
| 1 | miR-146b | MIMAT0002809 | hsa-miR-146b-5p | up |
| 2 | miR-221 | MIMAT0000278 | hsa-miR-221-3p | up |
| 3 | miR-222 | MIMAT0000279 | hsa-miR-222-3p | up |

|  | **Supplemental Dataset 5.** | | | | | |
| --- | --- | --- | --- | --- | --- | --- |
|  | miRNA_ID | miRBase21_Accession | miRBase21_ID | FC | P-value | Label |
| 1 | miR-146b | MIMAT0002809 | hsa-miR-146b-5p | 59.7 | 0005 | up |
| 2 | miR-221 | MIMAT0000278 | hsa-miR-221-3p | 20.7 | 0005 | up |
| 3 | miR-222 | MIMAT0000279 | hsa-miR-222-3p | 13.9 | 0005 | up |
| 4 | miR-181b | MIMAT0000257 | hsa-miR-181b-5p | 2 | 0022 | up |

|  | **Supplemental Dataset 6.** | | | |
| --- | --- | --- | --- | --- |
|  | miRNA_ID | miRBase21_Accession | miRBase21_ID | Label |
| 1 | miR-146b | MIMAT0002809 | hsa-miR-146b-5p | up |
| 2 | miR-221 | MIMAT0000278 | hsa-miR-221-3p | up |
| 3 | miR-222 | MIMAT0000279 | hsa-miR-222-3p | up |

| \|  \| **Supplemental Dataset 7.** \| \| \| \| \| --- \| --- \| --- \| --- \| --- \| \|  \| miRNA_ID \| miRBase21_Accession \| miRBase21_ID \| Label \| \| 1 \| mir-146b \| MIMAT0002809 \| hsa-miR-146b-5p \| up \| \| 2 \| mir-221 \| MIMAT0000278 \| hsa-miR-221-3p \| up \| \| 3 \| mir-222 \| MIMAT0000279 \| hsa-miR-222-3p \| up \| \| 4 \| mir-146a \| MIMAT0000449 \| hsa-miR-146a-5p \| up \| \| 5 \| mir-34a \| MIMAT0000255 \| hsa-miR-34a-5p \| up \| \| 6 \| mir-21 \| MIMAT0000076 \| hsa-miR-21-5p \| up \| \| 7 \| mir-31 \| MIMAT0000089 \| hsa-miR-31-5p \| up \| \| 8 \| mir-375 \| MIMAT0000728 \| hsa-miR-375 \| up \| \| 9 \| mir-181b \| MIMAT0000257 \| hsa-miR-181b-5p \| up \| \| 10 \| miR-100 \| MIMAT0000098 \| hsa-miR-100-5p \| down \| \| 11 \| mir-199b \| MIMAT0000263 \| hsa-miR-199b-5p \| down \| \| 12 \| mir-638 \| MIMAT0003308 \| hsa-miR-638 \| down \| \| 13 \| mir-451 \| MIMAT0001631 \| hsa-miR-451a \| down \| \| 14 \| mir-7 \| MIMAT0000252 \| hsa-miR-7-5p \| down \| |  |
| --- | --- | --- | --- | --- | --- | --- | --- | --- | --- | --- | --- | --- | --- | --- | --- | --- | --- | --- | --- | --- | --- | --- | --- | --- | --- | --- | --- | --- | --- | --- | --- | --- | --- | --- | --- | --- | --- | --- | --- | --- | --- | --- | --- | --- | --- | --- | --- | --- | --- | --- | --- | --- | --- | --- | --- | --- | --- | --- | --- | --- | --- | --- | --- | --- | --- | --- | --- | --- | --- | --- | --- | --- | --- | --- | --- | --- | --- | --- | --- | --- | --- |

|  | **Supplemental Dataset 8.** | | | |  |
| --- | --- | --- | --- | --- | --- |
|  | miRNA_ID | miRBase21_Accession | miRBase21_ID | Label |  |
| 1 | miR-146b | MIMAT0002809 | hsa-miR-146b-5p | up |  |
| 2 | miR-221 | MIMAT0000278 | hsa-miR-221-3p | up |  |
| 3 | miR-222 | MIMAT0000279 | hsa-miR-222-3p | up |  |
| 4 | miR-155 | MIMAT0000646 | hsa-miR-155-5p | up |  |
| 5 | miR-31 | MIMAT0000089 | hsa-miR-31-5p | up |  |
| 6 | miR-153 | MIMAT0000439 | hsa-miR-153-3p | down |  |
| 7 | miR-448 | MIMAT0001532 | hsa-miR-448 | down |  |
| 8 | miR-325 | MIMAT0000771 | hsa-miR-325 | down |  |
| 9 | miR-382 | MIMAT0000737 | hsa-miR-382-5p | down |  |
| 10 | miR-495 | MIMAT0002817 | hsa-miR-495-3p | down |  |
| 11 | miR-34b** | MIMAT0000685 | hsa-miR-34b-5p | down |  |
|  |  | MIMAT0004676 | hsa-miR-34b-3p | down |  |
| 12 | miR-1 | MIMAT0000416 | hsa-miR-1-3p | down |  |
| 13 | miR-144 | MIMAT0000436 | hsa-miR-144-3p | down |  |
| 14 | miR-367 | MIMAT0000719 | hsa-miR-367-3p | down |  |
| 15 | miR-130b | MIMAT0000691 | hsa-miR-130b-3p | down |  |
| 16 | miR-138 | MIMAT0000430 | hsa-miR-138-5p | down |  |
|  | **two different mature miRNA sequences are reported with previous ID hsa-miR-34b by miRBase Tracker | | | | |

|  | **Supplemental Dataset 9.** | | | |
| --- | --- | --- | --- | --- |
|  | miRNA_ID | miRBase21_Accession | miRBase21_ID | Label |
| 1 | miR-15a | MIMAT0000068 | hsa-miR-15a-5p | up |
| 2 | miR-31* | MIMAT0004504 | hsa-miR-31-3p | up |
| 3 | miR-1274a | MIMAT0005927 | hsa-miR-1274a | up |
| 4 | miR-15a* | MIMAT0004488 | hsa-miR-15a-3p | up |
| 5 | miR-551b | MIMAT0003233 | hsa-miR-551b-3p | up |
| 6 | miR-34a* | MIMAT0004557 | hsa-miR-34a-3p | up |
| 7 | miR-221* | MIMAT0004568 | hsa-miR-221-5p | up |
| 8 | miR-34b* | MIMAT0000685 | hsa-miR-34b-5p | up |
| 9 | miR-34a | MIMAT0000255 | hsa-miR-34a-5p | up |
| 10 | miR-222 | MIMAT0000279 | hsa-miR-222-3p | up |
| 11 | miR-221 | MIMAT0000278 | hsa-miR-221-3p | up |
| 12 | miR-146b-5p | MIMAT0002809 | hsa-miR-146b-5p | up |
| 13 | miR-873 | MIMAT0004953 | hsa-miR-873-5p | down |
| 14 | miR-876-3p | MIMAT0004925 | hsa-miR-876-3p | down |
| 15 | miR-7-2* | MIMAT0004554 | hsa-miR-7-2-3p | down |
| 16 | miR-7 | MIMAT0000252 | hsa-miR-7-5p | down |
| 17 | miR-30a | MIMAT0000087 | hsa-miR-30a-5p | down |
| 18 | miR-144* | MIMAT0004600 | hsa-miR-144-5p | down |

|  | **Supplemental Dataset 10.** | | | | | | |
| --- | --- | --- | --- | --- | --- | --- | --- |
|  | miRNA_ID | miRBase21_Accession | miRBase21_ID | FC (log2) | P-value | Label |  |
| 1 | miR-146b-5p | MIMAT0002809 | hsa-miR-146b-5p | 7,69 | 0,0151 | up |  |
| 2 | miR-221 | MIMAT0000278 | hsa-miR-221-3p | 4,86 | 0,0069 | up |  |
| 3 | miR-222 | MIMAT0000279 | hsa-miR-222-3p | 4,08 | 0,0051 | up |  |
| 4 | miR-34a | MIMAT0000255 | hsa-miR-34a-5p | 3,91 | 0,0068 | up |  |
| 5 | miR-375 | MIMAT0000728 | hsa-miR-375 | 3,49 | 0,0394 | up |  |
| 6 | miR-31 | MIMAT0000089 | hsa-miR-31-5p | 2,75 | 0,0178 | up |  |
| 7 | miR-181a-2* | MIMAT0004558 | hsa-miR-181a-2-3p | 2,52 | 0,0076 | up |  |
| 8 | miR-21 | MIMAT0000076 | hsa-miR-21-5p | 2,4 | 0,002 | up |  |
| 9 | miR-181c | MIMAT0000258 | hsa-miR-181c-5p | 2,27 | 0,0096 | up |  |
| 10 | miR-182 | MIMAT0000259 | hsa-miR-182-5p | 1,35 | 0,0411 | up |  |
| 11 | miR-130a | MIMAT0000425 | hsa-miR-130a-3p | -1,42 | 0,026 | down |  |
| 12 | miR-126 | MIMAT0000445 | hsa-miR-126-3p | -1,95 | 0,0145 | down |  |
| 13 | miR-374b | MIMAT0004955 | hsa-miR-374b-5p | -2,25 | 0,0279 | down |  |
| 14 | miR-486-5p | MIMAT0002177 | hsa-miR-486-5p | -2,87 | 0,0411 | down |  |
| 15 | miR-335 | MIMAT0000765 | hsa-miR-335-5p | -3,54 | 0,0157 | down |  |

|  | **Supplemental Dataset 11.** | | | | |
| --- | --- | --- | --- | --- | --- |
|  | miRNA_ID | miRBase21_Accession | miRBase21_ID | FC | Label |
| 1 | hsa-miR-551b | MIMAT0003233 | hsa-miR-551b-3p | 30,3 | up |
| 2 | hsa-miR-146b | MIMAT0002809 | hsa-miR-146b-5p | 16,47 | up |
| 3 | hsa-miR-222 | MIMAT0000279 | hsa-miR-222-3p | 11,47 | up |
| 4 | hsa-miR-221 | MIMAT0000278 | hsa-miR-221-3p | 11,14 | up |
| 5 | hsa-miR-31 | MIMAT0000089 | hsa-miR-31-5p | 5,86 | up |
| 6 | hsa-miR-96 | MIMAT0000095 | hsa-miR-96-5p | 4,78 | up |
| 7 | hsa-miR-21 | MIMAT0000076 | hsa-miR-21-5p | 3,08 | up |
| 8 | hsa-miR-135b | MIMAT0000758 | hsa-miR-135b-5p | 2,09 | up |
| 9 | hsa-miR-181b | MIMAT0000257 | hsa-miR-181b-5p | 2,06 | up |
| 10 | hsa-miR-7 | MIMAT0000252 | hsa-miR-7-5p | 0,17 | down |
| 11 | hsa-miR-138 | MIMAT0000430 | hsa-miR-138-5p | 0,21 | down |
| 12 | hsa-miR-150 | MIMAT0000451 | hsa-miR-150-5p | 0,25 | down |
| 13 | hsa-miR-1275 | MIMAT0005929 | hsa-miR-1275 | 0,37 | down |
| 14 | hsa-miR-199–5p | MIMAT0000263 | hsa-miR-199b-5p | 0,37 | down |

|  | **Supplemental Dataset 12.** | | | |
| --- | --- | --- | --- | --- |
|  | miRNA_ID | miRBase21_Accession | miRBase21_ID | Label |
| 1 | hsa-miR-146b-5p | MIMAT0002809 | hsa-miR-146b-5p | up |
| 2 | hsa-miR-375 | MIMAT0000728 | hsa-miR-375 | up |
| 3 | hsa-miR-146b-3p | MIMAT0004766 | hsa-miR-146b-3p | up |
| 4 | hsa-miR-551b | MIMAT0003233 | hsa-miR-551b-3p | up |
| 5 | hsa-miR-222 | MIMAT0000279 | hsa-miR-222-3p | up |
| 6 | hsa-miR-221 | MIMAT0000278 | hsa-miR-221-3p | up |
| 7 | hsa-miR-181a-2-3p | MIMAT0004558 | hsa-miR-181a-2-3p | up |
| 8 | hsa-miR-21 | MIMAT0000076 | hsa-miR-21-5p | up |
| 9 | hsa-miR-15a-3p | MIMAT0004488 | hsa-miR-15a-3p | up |
| 10 | hsa-miR-34a | MIMAT0000255 | hsa-miR-34a-5p | up |
| 11 | hsa-miR-451 | MIMAT0001631 | hsa-miR-451a | down |
| 12 | hsa-miR-144-5p | MIMAT0004600 | hsa-miR-144-5p | down |
| 13 | hsa-miR-138 | MIMAT0000430 | hsa-miR-138-5p | down |
| 14 | hsa-miR-204 | MIMAT0000265 | hsa-miR-204-5p | down |
| 15 | hsa-miR-1179 | MIMAT0005824 | hsa-miR-1179 | down |
| 16 | hsa-miR-219-5p | MIMAT0000276 | hsa-miR-219a-5p | down |

|  | **Supplemental Dataset 13_1.** | | | | | |
| --- | --- | --- | --- | --- | --- | --- |
|  | miRNA_ID | miRBase21_Accession | miRBase21_ID | FC | Paired FDR | Label |
| 1 | hsa-miR-146b-3p | MIMAT0004766 | hsa-miR-146b-3p | 10,81 | 0,031 | up |
| 2 | hsa-miR-146b-5p | MIMAT0002809 | hsa-miR-146b-5p | 10,54 | 0,027 | up |
| 3 | hsa-miR-891a | MIMAT0004902 | hsa-miR-891a-5p | 7,68 | 0,031 | up |
| 4 | hsa-miR-551b-3p | MIMAT0003233 | hsa-miR-551b-3p | 6,53 | 0,025 | up |
| 5 | hsa-miR-187-3p | MIMAT0000262 | hsa-miR-187-3p | 5,1 | 0,031 | up |
| 6 | hsa-miR-221-5p | MIMAT0004568 | hsa-miR-221-5p | 4,57 | 0,024 | up |
| 7 | hsa-miR-147b | MIMAT0004928 | hsa-miR-147b | 4,53 | 0,036 | up |
| 8 | hsa-miR-21-3p | MIMAT0004494 | hsa-miR-21-3p | 4,04 | 0,031 | up |
| 9 | hsa-miR-181b-3p | MIMAT0022692 | hsa-miR-181b-3p | 3,91 | 0,02 | up |
| 10 | hsa-miR-222-5p | MIMAT0004569 | hsa-miR-222-5p | 3,63 | 0,039 | up |
| 11 | hsa-miR-182-5p | MIMAT0000259 | hsa-miR-182-5p | 2,63 | 0,031 | up |
| 12 | hsa-miR-33b-5p | MIMAT0003301 | hsa-miR-33b-5p | 2,55 | 0,01 | up |
| 13 | hsa-miR-203a | MIMAT0000264 | hsa-miR-203a-3p | 2,49 | 0,031 | up |
| 14 | hsa-miR-183-5p | MIMAT0000261 | hsa-miR-183-5p | 2,48 | 0,024 | up |
| 15 | hsa-miR-589-5p | MIMAT0004799 | hsa-miR-589-5p | 2,39 | 0,049 | up |
| 16 | hsa-miR-3913-5p | MIMAT0018187 | hsa-miR-3913-5p | 2,29 | 0,031 | up |
| 17 | hsa-miR-135b-5p | MIMAT0000758 | hsa-miR-135b-5p | 1,84 | 0,024 | up |
| 18 | hsa-miR-744-3p | MIMAT0004946 | hsa-miR-744-3p | 1,49 | 0,024 | up |
| 19 | hsa-miR-17-3p | MIMAT0000071 | hsa-miR-17-3p | 1,35 | 0,024 | up |
| 20 | hsa-miR-574-3p | MIMAT0003239 | hsa-miR-574-3p | 0,65 | 0,05 | down |
| 21 | hsa-miR-3200-3p | MIMAT0015085 | hsa-miR-3200-3p | 0,62 | 0,012 | down |
| 22 | hsa-let-7d-5p | MIMAT0000065 | hsa-let-7d-5p | 0,61 | 0,031 | down |
| 23 | hsa-miR-130a-3p | MIMAT0000425 | hsa-miR-130a-3p | 0,6 | 0,031 | down |
| 24 | hsa-miR-100-5p | MIMAT0000098 | hsa-miR-100-5p | 0,53 | 0,031 | down |
| 25 | hsa-miR-195-5p | MIMAT0000461 | hsa-miR-195-5p | 0,51 | 0,049 | down |
| 26 | hsa-miR-585 | MIMAT0003250 | hsa-miR-585-3p | 0,49 | 0,031 | down |
| 27 | hsa-miR-532-5p | MIMAT0002888 | hsa-miR-532-5p | 0,49 | 0,024 | down |
| 28 | hsa-miR-455-3p | MIMAT0004784 | hsa-miR-455-3p | 0,48 | 0,031 | down |
| 29 | hsa-miR-138-5p | MIMAT0000430 | hsa-miR-138-5p | 0,47 | 0,031 | down |
| 30 | hsa-miR-1249 | MIMAT0005901 | hsa-miR-1249-3p | 0,43 | 0,043 | down |
| 31 | hsa-miR-20b-5p | MIMAT0001413 | hsa-miR-20b-5p | 0,4 | 0,031 | down |
| 32 | hsa-miR-145-5p | MIMAT0000437 | hsa-miR-145-5p | 0,4 | 0,024 | down |
| 33 | hsa-miR-1180 | MIMAT0005825 | hsa-miR-1180-3p | 0,39 | 0,024 | down |
| 34 | hsa-miR-652-3p | MIMAT0003322 | hsa-miR-652-3p | 0,38 | 0,049 | down |
| 35 | hsa-miR-1291 | MIMAT0005881 | hsa-miR-1291 | 0,35 | 0,01 | down |
| 36 | hsa-miR-451a | MIMAT0001631 | hsa-miR-451a | 0,31 | 0,031 | down |
| 37 | hsa-miR-486-5p | MIMAT0002177 | hsa-miR-486-5p | 0,31 | 0,031 | down |
| 38 | hsa-miR-138-1-3p | MIMAT0004607 | hsa-miR-138-1-3p | 0,31 | 0,017 | down |
| 39 | hsa-miR-144-5p | MIMAT0004600 | hsa-miR-144-5p | 0,28 | 0,017 | down |
| 40 | hsa-miR-486-3p | MIMAT0004762 | hsa-miR-486-3p | 0,23 | 0,002 | down |
| 41 | hsa-miR-873-5p | MIMAT0004953 | hsa-miR-873-5p | 0,22 | 0,024 | down |
| 42 | hsa-miR-204-5p | MIMAT0000265 | hsa-miR-204-5p | 0,2 | 0,033 | down |
| 43 | hsa-miR-7-2-3p | MIMAT0004554 | hsa-miR-7-2-3p | 0,17 | 0,034 | down |
| 44 | hsa-miR-1179 | MIMAT0005824 | hsa-miR-1179 | 0,14 | 0,031 | down |

|  | **Supplemental Dataset 13_2.** | | | | | | |
| --- | --- | --- | --- | --- | --- | --- | --- |
|  | miRNA_ID | miRBase21_Accession | miRBase21_ID | FC | FDR | Label |  |
| 1 | hsa-miR-146b-5p | MIMAT0004766 | hsa-miR-146b-3p | 20,69 | 0,001 | up |  |
| 2 | hsa-miR-146b-3p | MIMAT0002809 | hsa-miR-146b-5p | 17,47 | 0,001 | up |  |
| 3 | hsa-miR-551b-3p | MIMAT0003233 | hsa-miR-551b-3p | 7 | 0,002 | up |  |
| 4 | hsa-miR-891a | MIMAT0004902 | hsa-miR-891a-5p | 5,92 | 0,027 | up |  |
| 5 | hsa-miR-187-3p | MIMAT0000262 | hsa-miR-187-3p | 5,58 | 0,004 | up |  |
| 6 | hsa-miR-221-5p | MIMAT0004568 | hsa-miR-221-5p | 5,03 | 0,001 | up |  |
| 7 | hsa-miR-222-5p | MIMAT0004569 | hsa-miR-222-5p | 3,51 | 0,003 | up |  |
| 8 | hsa-miR-33b-5p | MIMAT0003301 | hsa-miR-33b-5p | 3,44 | 0,003 | up |  |
| 9 | hsa-miR-181b-3p | MIMAT0022692 | hsa-miR-181b-3p | 2,94 | 0,005 | up |  |
| 10 | hsa-miR-182-5p | MIMAT0000259 | hsa-miR-182-5p | 2,9 | 0,003 | up |  |
| 11 | hsa-miR-203a | MIMAT0000264 | hsa-miR-203a-3p | 2,63 | 0,007 | up |  |
| 12 | hsa-miR-183-5p | MIMAT0000261 | hsa-miR-183-5p | 2,14 | 0,008 | up |  |
| 13 | hsa-miR-135b-5p | MIMAT0000758 | hsa-miR-135b-5p | 1,91 | 0 | up |  |
| 14 | hsa-miR-744-3p | MIMAT0004946 | hsa-miR-744-3p | 1,85 | 0,003 | up |  |
| 15 | hsa-let-7d-5p | MIMAT0000065 | hsa-let-7d-5p | 0,53 | 0,003 | down |  |
| 16 | hsa-miR-195-5p | MIMAT0000461 | hsa-miR-195-5p | 0,52 | 0,01 | down |  |
| 17 | hsa-miR-574-3p | MIMAT0003239 | hsa-miR-574-3p | 0,52 | 0,003 | down |  |
| 18 | hsa-miR-455-3p | MIMAT0004784 | hsa-miR-455-3p | 0,42 | 0,001 | down |  |
| 19 | hsa-miR-532-5p | MIMAT0002888 | hsa-miR-532-5p | 0,41 | 0,003 | down |  |
| 20 | hsa-miR-1291 | MIMAT0005881 | hsa-miR-1291 | 0,4 | 0,01 | down |  |
| 21 | hsa-miR-451a | MIMAT0001631 | hsa-miR-451a | 0,32 | 0,019 | down |  |
| 22 | hsa-miR-20b-5p | MIMAT0001413 | hsa-miR-20b-5p | 0,32 | 0,009 | down |  |
| 23 | hsa-miR-585 | MIMAT0003250 | hsa-miR-585-3p | 0,3 | 0,01 | down |  |
| 24 | hsa-miR-145-5p | MIMAT0000437 | hsa-miR-145-5p | 0,3 | 0 | down |  |
| 25 | hsa-miR-144-5p | MIMAT0004600 | hsa-miR-144-5p | 0,26 | 0,006 | down |  |
| 26 | hsa-miR-204-5p | MIMAT0000265 | hsa-miR-204-5p | 0,24 | 0,019 | down |  |
| 27 | hsa-miR-873-5p | MIMAT0004953 | hsa-miR-873-5p | 0,23 | 0,027 | down |  |
| 28 | hsa-miR-1249 | MIMAT0005901 | hsa-miR-1249-3p | 0,23 | 0,001 | down |  |
| 29 | hsa-miR-138-1-3p | MIMAT0004607 | hsa-miR-138-1-3p | 0,23 | 0 | down |  |
| 30 | hsa-miR-486-3p | MIMAT0002177 | hsa-miR-486-5p | 0,17 | 0 | down |  |
| 31 | hsa-miR-1179 | MIMAT0005824 | hsa-miR-1179 | 0,13 | 0,008 | down |  |
| 32 | hsa-miR-7-2-3p | MIMAT0004554 | hsa-miR-7-2-3p | 0,13 | 0,006 | down |  |

|  | **Supplemental Dataset 13_3.** | | | | |
| --- | --- | --- | --- | --- | --- |
|  | miRNA_ID | miRBase21_Accession | miRBase21_ID | FC | Label |
| 1 | hsa-miR-146b-5p | MIMAT0002809 | hsa-miR-146b-5p | 4,7 | up |
| 2 | hsa-miR-182-5p | MIMAT0000259 | hsa-miR-182-5p | 1,7 | up |
| 3 | hsa-miR-203a | MIMAT0000264 | hsa-miR-203a-3p | 1,61 | up |
| 4 | hsa-miR-744-3p | MIMAT0004946 | hsa-miR-744-3p | 1,43 | up |
| 5 | hsa-miR-551b-3p | MIMAT0003233 | hsa-miR-551b-3p | 1,29 | up |
| 6 | hsa-miR-21-3p | MIMAT0004494 | hsa-miR-21-3p | 1,11 | up |
| 7 | hsa-miR-574-3p | MIMAT0003239 | hsa-miR-574-3p | 0,99 | down |
| 8 | hsa-miR-532-5p | MIMAT0002888 | hsa-miR-532-5p | 0,95 | down |
| 9 | hsa-miR-195-5p | MIMAT0000461 | hsa-miR-195-5p | 0,9 | down |
| 10 | hsa-miR-455-3p | MIMAT0004784 | hsa-miR-455-3p | 0,85 | down |
| 11 | hsa-let-7d-5p | MIMAT0000065 | hsa-let-7d-5p | 0,75 | down |
| 12 | hsa-miR-145-5p | MIMAT0000437 | hsa-miR-145-5p | 0,73 | down |
| 13 | hsa-miR-652-3p | MIMAT0003322 | hsa-miR-652-3p | 0,68 | down |
| 14 | hsa-miR-20b-5p | MIMAT0001413 | hsa-miR-20b-5p | 0,64 | down |
| 15 | hsa-miR-130a-3p | MIMAT0000425 | hsa-miR-130a-3p | 0,63 | down |
| 16 | hsa-miR-100-5p | MIMAT0000098 | hsa-miR-100-5p | 0,51 | down |
| 17 | hsa-miR-138-5p | MIMAT0000430 | hsa-miR-138-5p | 0,4 | down |
| 18 | hsa-miR-7-2-3p | MIMAT0004554 | hsa-miR-7-2-3p | 0,37 | down |
| 19 | hsa-miR-144-5p | MIMAT0004600 | hsa-miR-144-5p | 0,31 | down |
| 20 | hsa-miR-486-5p | MIMAT0002177 | hsa-miR-486-5p | 0,28 | down |
| 21 | hsa-miR-451a | MIMAT0001631 | hsa-miR-451a | 0,27 | down |
| 22 | hsa-miR-204-5p | MIMAT0000265 | hsa-miR-204-5p | 0,11 | down |

|  | **Supplemental Dataset 14_1.** | | | | | |
| --- | --- | --- | --- | --- | --- | --- |
|  | miRNA_ID | miRBase21_Accession | miRBase21_ID | FC | W.pval.BH.paired | Label |
| 1 | miR-146b-5p | MIMAT0002809 | hsa-miR-146b-5p | 46,22 | 1,66E-08 | up |
| 2 | miR-146b-3p | MIMAT0004766 | hsa-miR-146b-3p | 41,76 | 1,66E-08 | up |
| 3 | miR-221-3p | MIMAT0000278 | hsa-miR-221-3p | 10,73 | 1,66E-08 | up |
| 4 | miR-222-3p | MIMAT0000279 | hsa-miR-222-3p | 9,06 | 1,66E-08 | up |
| 5 | miR-375 | MIMAT0000728 | hsa-miR-375 | 7,84 | 6,02E-08 | up |
| 6 | miR-21-5p | MIMAT0000076 | hsa-miR-21-5p | 3,72 | 3,26E-08 | up |
| 7 | miR-34a-5p | MIMAT0000255 | hsa-miR-34a-5p | 3,49 | 2,32E-08 | up |
| 8 | miR-31-5p | MIMAT0000089 | hsa-miR-31-5p | 3,44 | 7,89E-07 | up |
| 9 | miR-181a-2-3p | MIMAT0004558 | hsa-miR-181a-2-3p | 2,64 | 4,67E-08 | up |
| 10 | miR-181b-5p | MIMAT0000257 | hsa-miR-181b-5p | 2,52 | 9,59E-08 | up |
| 11 | miR-181a-1-5p | MIMAT0000256 | hsa-miR-181a-1-5p | 2,12 | 3,98E-07 | up |
| 12 | miR-3065-3p | MIMAT0015378 | hsa-miR-3065-3p | 2,09 | 1,65E-05 | up |
| 13 | miR-204-5p | MIMAT0000265 | hsa-miR-204-5p | -11,81 | 1,14E-05 | down |
| 14 | miR-486-5p | MIMAT0002177 | hsa-miR-486-5p | -8,66 | 2,51E-08 | down |
| 15 | miR-199b-5p | MIMAT0000263 | hsa-miR-199b-5p | -8,63 | 2,51E-08 | down |
| 16 | miR-144-5p | MIMAT0004600 | hsa-miR-144-5p | -6,53 | 2,32E-08 | down |
| 17 | miR-451a | MIMAT0001631 | hsa-miR-451a | -6,43 | 2,32E-08 | down |
| 18 | miR-9-5p | MIMAT0000441 | hsa-miR-9-5p | -6,31 | 3,29E-08 | down |
| 19 | miR-144-3p | MIMAT0000436 | hsa-miR-144-3p | -4,38 | 2,32E-08 | down |
| 20 | miR-199b-3p | MIMAT0004563 | hsa-miR-199b-3p | -3,84 | 9,59E-08 | down |
| 21 | miR-199a-3p | MIMAT0000232 | hsa-miR-199a-3p | -3,83 | 9,59E-08 | down |
| 22 | miR-379-5p | MIMAT0000733 | hsa-miR-379-5p | -3,38 | 4,05E-05 | down |
| 23 | miR-138-5p | MIMAT0000430 | hsa-miR-138-5p | -3,37 | 4,02E-08 | down |
| 24 | miR-345-5p | MIMAT0000772 | hsa-miR-345-5p | -3,07 | 0,000103 | down |
| 25 | miR-652-3p | MIMAT0003322 | hsa-miR-652-3p | -2,93 | 1,66E-08 | down |
| 26 | miR-199a-5p | MIMAT0000231 | hsa-miR-199a-5p | -2,74 | 7,77E-07 | down |
| 27 | miR-195-5p | MIMAT0000461 | hsa-miR-195-5p | -2,73 | 4,67E-08 | down |
| 28 | miR-127-3p | MIMAT0000446 | hsa-miR-127-3p | -2,50 | 0,000218 | down |
| 29 | miR-152-3p | MIMAT0000438 | hsa-miR-152-3p | -2,36 | 4,82E-08 | down |
| 30 | miR-134-5p | MIMAT0000447 | hsa-miR-134-5p | -2,27 | 0,000267 | down |
| 31 | miR-139-3p | MIMAT0004552 | hsa-miR-139-3p | -2,27 | 1,66E-08 | down |
| 32 | miR-139-5p | MIMAT0000250 | hsa-miR-139-5p | -2,25 | 2,81E-08 | down |
| 33 | miR-708-5p | MIMAT0004926 | hsa-miR-708-5p | -2,17 | 5,91E-08 | down |
| 34 | miR-100-5p | MIMAT0000098 | hsa-miR-100-5p | -2,16 | 1,16E-07 | down |
| 35 | miR-10a-5p | MIMAT0000253 | hsa-miR-10a-5p | -2,15 | 3,83E-07 | down |
| 36 | miR-145-5p | MIMAT0000437 | hsa-miR-145-5p | -2,13 | 9,82E-08 | down |
| 37 | miR-28-5p | MIMAT0000085 | hsa-miR-28-5p | -2,12 | 2,32E-08 | down |
| 38 | miR-223-3p | MIMAT0000280 | hsa-miR-223-3p | -2,11 | 1,49E-06 | down |
| 39 | miR-218-5p | MIMAT0000275 | hsa-miR-218-5p | -2,10 | 7,89E-07 | down |
| 40 | miR-28-3p | MIMAT0004502 | hsa-miR-28-3p | -2,06 | 2,32E-08 | down |
| 41 | miR-150-5p | MIMAT0000451 | hsa-miR-150-5p | -2,01 | 1,98E-05 | down |

|  | **Supplemental Dataset 14_2.** | | | | | |
| --- | --- | --- | --- | --- | --- | --- |
|  | miRNA_ID | miRBase21_Accession | miRBase21_ID | FC | W.pval.BH.unpaired | Label |
| 1 | miR-146b-3p | MIMAT0004766 | hsa-miR-146b-3p | 60,12 | 3,24E-22 | up |
| 2 | miR-146b-5p | MIMAT0002809 | hsa-miR-146b-5p | 55,98 | 3,79E-22 | up |
| 3 | miR-221-3p | MIMAT0000278 | hsa-miR-221-3p | 9,19 | 5,9E-25 | up |
| 4 | miR-222-3p | MIMAT0000279 | hsa-miR-222-3p | 8,40 | 2,35E-24 | up |
| 5 | miR-375 | MIMAT0000728 | hsa-miR-375 | 8,08 | 1,56E-13 | up |
| 6 | miR-34a-5p | MIMAT0000255 | hsa-miR-34a-5p | 3,97 | 9,92E-28 | up |
| 7 | miR-21-5p | MIMAT0000076 | hsa-miR-21-5p | 3,45 | 6,51E-18 | up |
| 8 | miR-31-5p | MIMAT0000089 | hsa-miR-31-5p | 3,44 | 3,95E-11 | up |
| 9 | miR-181b-5p | MIMAT0000257 | hsa-miR-181b-5p | 2,64 | 3E-20 | up |
| 10 | miR-181a-2-3p | MIMAT0004558 | hsa-miR-181a-2-3p | 2,55 | 6,45E-20 | up |
| 11 | miR-181a-1-5p | MIMAT0000256 | hsa-miR-181a-1-5p | 2,16 | 4,85E-19 | up |
| 12 | miR-204-5p | MIMAT0000265 | hsa-miR-204-5p | -9,33 | 2,75E-19 | down |
| 13 | miR-486-5p | MIMAT0002177 | hsa-miR-486-5p | -7,87 | 5,9E-25 | down |
| 14 | miR-144-5p | MIMAT0004600 | hsa-miR-144-5p | -6,38 | 5,9E-25 | down |
| 15 | miR-451a | MIMAT0001631 | hsa-miR-451a | -6,36 | 5,9E-25 | down |
| 16 | miR-199b-5p | MIMAT0000263 | hsa-miR-199b-5p | -5,50 | 1,85E-19 | down |
| 17 | miR-9-5p | MIMAT0000441 | hsa-miR-9-5p | -5,43 | 5,9E-25 | down |
| 18 | miR-144-3p | MIMAT0000436 | hsa-miR-144-3p | -5,08 | 2,53E-21 | down |
| 19 | miR-138-5p | MIMAT0000430 | hsa-miR-138-5p | -3,43 | 2,4E-20 | down |
| 20 | miR-379-5p | MIMAT0000733 | hsa-miR-379-5p | -3,24 | 8,09E-14 | down |
| 21 | miR-199b-3p | MIMAT0004563 | hsa-miR-199b-3p | -3,07 | 4,36E-16 | down |
| 22 | miR-199a-3p | MIMAT0000232 | hsa-miR-199a-3p | -3,06 | 4,15E-16 | down |
| 23 | miR-652-3p | MIMAT0003322 | hsa-miR-652-3p | -2,82 | 1,22E-23 | down |
| 24 | miR-195-5p | MIMAT0000461 | hsa-miR-195-5p | -2,59 | 7,71E-23 | down |
| 25 | miR-345-5p | MIMAT0000772 | hsa-miR-345-5p | -2,53 | 1,56E-09 | down |
| 26 | miR-152-3p | MIMAT0000438 | hsa-miR-152-3p | -2,45 | 3,71E-19 | down |
| 27 | miR-134-5p | MIMAT0000447 | hsa-miR-134-5p | -2,37 | 4,75E-09 | down |
| 28 | miR-127-3p | MIMAT0000446 | hsa-miR-127-3p | -2,31 | 7,5E-09 | down |
| 29 | miR-708-5p | MIMAT0004926 | hsa-miR-708-5p | -2,30 | 5,95E-17 | down |
| 30 | miR-139-3p | MIMAT0004552 | hsa-miR-139-3p | -2,19 | 1,2E-16 | down |
| 31 | miR-199a-5p | MIMAT0000231 | hsa-miR-199a-5p | -2,18 | 5,98E-10 | down |
| 32 | miR-100-5p | MIMAT0000098 | hsa-miR-100-5p | -2,18 | 5,6E-21 | down |
| 33 | miR-3607-3p | MIMAT0017985 | hsa-miR-3607-3p | -2,14 | 3,61E-05 | down |
| 34 | miR-455-3p | MIMAT0004784 | hsa-miR-455-3p | -2,12 | 1,17E-10 | down |
| 35 | miR-150-5p | MIMAT0000451 | hsa-miR-150-5p | -2,11 | 3,45E-09 | down |
| 36 | miR-223-3p | MIMAT0000280 | hsa-miR-223-3p | -2,04 | 1,03E-11 | down |
| 37 | miR-218-5p | MIMAT0000275 | hsa-miR-218-5p | -2,02 | 5,75E-17 | down |

|  | **Supplemental Dataset 15.** | | | |
| --- | --- | --- | --- | --- |
|  | miRNA_ID | miRBase21_Accession | miRBase21_ID | Label |
| 1 | hsa-miR-21-5p | MIMAT0000076 | hsa-miR-21-5p | up |
| 2 | hsa-miR-146b-3p | MIMAT0004766 | hsa-miR-146b-3p | up |
| 3 | hsa-miR-146b-5p | MIMAT0002809 | hsa-miR-146b-5p | up |
| 4 | hsa-miR-221-5p | MIMAT0004568 | hsa-miR-221-5p | up |
| 5 | hsa-miR-21-3p | MIMAT0004494 | hsa-miR-21-3p | up |
| 6 | hsa-miR-34a-5p | MIMAT0000255 | hsa-miR-34a-5p | up |
| 7 | hsa-miR-221-3p | MIMAT0000278 | hsa-miR-221-3p | up |
| 8 | hsa-miR-222-3p | MIMAT0000279 | hsa-miR-222-3p | up |
| 9 | hsa-miR-224-5p | MIMAT0000281 | hsa-miR-224-5p | up |
| 10 | hsa-miR-31-3p | MIMAT0004504 | hsa-miR-31-3p | up |
| 11 | hsa-miR-3687 | MIMAT0018115 | hsa-miR-3687 | down |
| 12 | hsa-miR-1179 | MIMAT0005824 | hsa-miR-1179 | down |
| 13 | hsa-miR-451a | MIMAT0001631 | hsa-miR-451a | down |
| 14 | hsa-miR-486-5p | MIMAT0002177 | hsa-miR-486-5p | down |
| 15 | hsa-miR-486-3p | MIMAT0004762 | hsa-miR-486-3p | down |
